# Neuroinvasiveness of the MR766 strain of Zika virus in IFNAR^-/-^ mice maps to prM residues conserved amongst African genotype viruses

**DOI:** 10.1371/journal.ppat.1009788

**Published:** 2021-07-26

**Authors:** Eri Nakayama, Fumihiro Kato, Shigeru Tajima, Shinya Ogawa, Kexin Yan, Kenta Takahashi, Yuko Sato, Tadaki Suzuki, Yasuhiro Kawai, Takuya Inagaki, Satoshi Taniguchi, Thuy T. Le, Bing Tang, Natalie A. Prow, Akihiko Uda, Takahiro Maeki, Chang-Kweng Lim, Alexander A. Khromykh, Andreas Suhrbier, Masayuki Saijo

**Affiliations:** 1 Department of Virology I, National Institute of Infectious Diseases, Tokyo, Japan; 2 QIMR Berghofer Medical Research Institute, Brisbane, Queensland, Australia; 3 Department of Applied Biological Chemistry, School of Agriculture and Life Sciences, The University of Tokyo, Tokyo, Japan; 4 Department of Pathology, National Institute of Infectious Diseases, Tokyo, Japan; 5 Management Department of Biosafety and Laboratory Animal, Division of Biosafety Control and Research, National Institute of Infectious Diseases, Tokyo, Japan; 6 Australian Infectious Disease Research Centre, GVN Center of Excellence, The University of Queensland and QIMR Berghofer Medical Research Institute, Brisbane, Queensland, Australia; 7 Department of Veterinary Science, National Institute of Infectious Diseases, Tokyo, Japan; 8 School of Chemistry and Molecular Biosciences, The University of Queensland, Brisbane, Queensland, Australia; Emory University, UNITED STATES

## Abstract

Zika virus (ZIKV) strains are classified into the African and Asian genotypes. The higher virulence of the African MR766 strain, which has been used extensively in ZIKV research, in adult IFNα/β receptor knockout (IFNAR^-/-^) mice is widely viewed as an artifact associated with mouse adaptation due to at least 146 passages in wild-type suckling mouse brains. To gain insights into the molecular determinants of MR766’s virulence, a series of genes from MR766 were swapped with those from the Asian genotype PRVABC59 isolate, which is less virulent in IFNAR^-/-^ mice. MR766 causes 100% lethal infection in IFNAR^-/-^ mice, but when the prM gene of MR766 was replaced with that of PRVABC59, the chimera MR/PR(prM) showed 0% lethal infection. The reduced virulence was associated with reduced neuroinvasiveness, with MR766 brain titers ≈3 logs higher than those of MR/PR(prM) after subcutaneous infection, but was not significantly different in brain titers of MR766 and MR/PR(prM) after intracranial inoculation. MR/PR(prM) also showed reduced transcytosis when compared with MR766 *in vitro*. The high neuroinvasiveness of MR766 in IFNAR^-/-^ mice could be linked to the 10 amino acids that differ between the prM proteins of MR766 and PRVABC59, with 5 of these changes affecting positive charge and hydrophobicity on the exposed surface of the prM protein. These 10 amino acids are highly conserved amongst African ZIKV isolates, irrespective of suckling mouse passage, arguing that the high virulence of MR766 in adult IFNAR^-/-^ mice is not the result of mouse adaptation.

## Author summary

Contemporary Asian genotype Zika virus (ZIKV) caused a large epidemic in the Americas and was associated with congenital abnormalities and neurological disease in adults, whereas African ZIKV has not been reported to cause neurological malformations in humans. Whether the lack of reported cases caused by African ZIKV simply reflects under-reporting remains unclear. Numerous studies have attempted to explain how and why these severe manifestations are restricted to ZIKV of Asian origin. Although several studies have reported that African ZIKV strains are more virulent in adult IFNα/β receptor knockout (IFNAR^-/-^) mice, the representative African ZIKV strain MR766 is believed to have artefactually high virulence in adult IFNAR^-/-^ mice due to its extensive passage history in suckling wild-type mouse brains. However, we provide evidence herein that the lethality of MR766 in the adult IFNAR^-/-^ mice is associated with 10 amino acid residues in the prM protein that are associated with an increased ability to cross the blood-brain barrier. These amino acids are highly conserved in African genotype viruses, irrespective of multiple passage history in suckling mouse brains, suggesting that high virulence is a legitimate characteristic of African ZIKVs.

## Introduction

The World Health Organization declared the outbreak of Zika virus (ZIKV) infection on the American continent a public health emergency of international concern in February 2016. This flavivirus induces a recognized spectrum of congenital neurological malformations (including but not restricted to microcephaly) termed Congenital Zika Syndrome (CZS) that will likely be associated with complex and life-long disabilities in children born to women infected with ZIKV during pregnancy [1,2].

ZIKV is an enveloped virus with ~11 kb positive-sense single-strand RNA genome. The ZIKV genome encodes a polyprotein that is post-translationally processed by cellular and viral proteases into three structural proteins, capsid (C), pre-membrane (prM) and envelope (E), and seven non-structural proteins: NS1, NS2A, NS2B, NS3, NS4A, NS4B and NS5. The immature virus is composed of 60 trimetric spikes of the prM and E proteins [3]. Maturation of the virus particles involves cleavage of the prM protein by the host protease furin. Although the cleavage of prM is required for exposure of the fusion loop, which is needed for viral membrane fusion with the host endosomal membrane, the process is usually incomplete, and complete maturation is not required for the acquisition of infectivity [4–6]. ZIKV virions contain a mixture of cleaved and uncleaved prM proteins [7,8], a phenomenon that is also seen in other flaviviruses [9–13]. The prM protein has been associated with the virulence of ZIKV [14–16] and other flaviviruses [4,17,18].

There are two genotypes or lineages of ZIKV, the African and the Asian. Two ZIKV isolates, MR766 and PRVABC59, have been used extensively as respective representatives of African and Asian genotype ZIKVs [19–21]. MR766 was isolated in 1947 from a febrile rhesus macaque in the Zika forest in Uganda [22,23], and PRVABC59 was isolated from the serum of a ZIKV-infected patient in Puerto Rico in 2015 [24]. Many studies have compared Asian genotype viruses with MR766 and have shown that MR766 was often more virulent than the Asian genotype viruses [19,20,25–27]. MR766 was passaged in wild-type suckling mice by intracranial (i.c.) injection [23] at least 146 times [28], with the resulting virus showing a highly neurovirulent phenotype in wild-type suckling mice after i.c. infection [23]. The results obtained using MR766 [19,25,29] and the relevance of studies using this mouse-adapted viral isolate are thus frequently brought into question [20,21,28,30–32]. Herein, we explore the molecular determinants that underpin the high virulence of MR766 in IFNα/β receptor knockout (IFNAR^-/-^) mice. The higher virulence of MR766 was associated with 10 amino acids in prM that are conserved in African ZIKV isolates, arguing that virulence in these mice was not the result of the mouse adaptation arising from the extensive passage in brains of wild-type suckling mice.

## Materials and methods

### Ethics statement

Mouse experiments were performed in accordance with the “Guidelines for Animal Experiments performed at National Institute of Infectious Diseases” under approval (nos. 116067, 116083, 119058, 119165, 120031, 120111, 120112 and 120156) from the Animal Welfare and Animal Care Committee of the National Institute of Infectious Diseases (NIID). All mice were bred and housed under specific-pathogen-free conditions. The mice were observed for symptoms, and their body weights were measured every day for 10 days post infection (dpi). Mice were euthanized using CO_2_ or cardiac puncture under isoflurane anesthesia when they reached ethically defined endpoints of excessively rapid body weight loss and/or persistent recumbent posture.

### Cells and viruses

Vero cells (strain 9013, JCRB9013) were obtained from the Japanese Collection of Research Bioresources Cell Bank (Osaka, Japan). Vero E6 cells (CRL1586), C6/36 cells (CRL1660), murine brain microvascular endothelial cells (bEnd.3 cells, CRL2299) and murine astrocyte cells (C8D1A cells, CRL2541) were obtained from the American Type Culture Collection (Manassas, VA). The cells were cultured according to the manufacturer’s instructions. PRVABC59 (Accession no. KU501215), which was isolated from a patient infected with ZIKV in Puerto Rico in 2015 [24], was kindly provided by Dr. Beth Bell of the US Center for Disease Control and Prevention. The amino acid position 330 in the E protein of PRVABC59 was a mixture of V and L as previously reported [33]. MR766 (MR766-NIID, Accession no. LC002520) was originally isolated from a rhesus monkey in Uganda in 1947 and then repeatedly passaged in mice and maintained at NIID, Japan [34]. MR766-NIID does not have the N-linked glycosylation at 154 in the E protein due to the T156I substitution [35,36].

The series of chimeric viruses, MR/PR(C), MR/PR(prM), MR/PR(E), MR/PR(NS5), MR/PR(NS4B/NS5), MR/PR(pr) and MR/PR(M) were constructed using rZIKV-MR766/pMW119-CMVP and primers (see S1 Table) as previously described [34]. Briefly, MR/PR(C), MR/PR(prM), MR/PR(E), MR/PR(NS5), MR/PR(pr) and MR/PR(M) were engineered by replacing MR766 genes coding for the C, prM, E, NS5, pr and M with corresponding genes of PRVABC59. The exact gene junctions were used to generate each chimera. For MR/PR(NS4B/NS5), the C terminal residues of NS4B (amino acids 187 to 251) and the entire NS5 gene were replaced from MR766 to PRVABC59. The cDNA of PRVABC59 was reverse-transcribed using Super Script III Reverse Transcriptase (Thermo Fisher Scientific, Tokyo, Japan) from RNA purified from the culture supernatant of PRVABC59-infected Vero cells. The fragments of PRVABC59 and the linearized rZIKV-MR766/pMW119-CMVP vector were amplified by using Q5 Hot Start High-Fidelity DNA Polymerase (New England BioLabs, Ipswich, MA) and the primers listed in S1 Table. The amplified fragments of PRVABC59 were inserted into the linearized rZIKV-MR766/pMW119-CMVP by using the In-Fusion Cloning system (Takara Bio USA, Inc., Mountain View, CA). The recombinant viruses were recovered from culture supernatants of Vero cells transfected with each recombinant clone. The recovered viruses were passaged in Vero cells to generate each recombinant virus stock. The complete nucleotide sequences of the recombinant virus from these stocks are provided for MR/PR(prM), MR/PR(C), MR/PR(E), MR/PR(NS5), MR/PR(NS4B/NS5), MR/PR(pr) or MR/PR(M) in GenBank Accession numbers LC571075, LC571076, LC571077, LC629061, LC629062, LC629063 or LC629064, respectively. The growth properties of each recombinant virus were determined as described previously [34]. All viruses were titered by plaque assay on Vero cells as described previously [34].

### Mice and ZIKV infection

IFNAR^-/-^ mice on a C57BL/6J background were generated and bred in-house at NIID. The gene knockout was undertaken in 129Sv/Ev-ES cells [37]. IFNAR^-/+^ and DNase2a^-/+^ mice were backcrossed eight times onto C57BL/6J mice [38] and were used to generate IFNAR^-/-^/DNase2a^-/-^ mice [39]. These mice were provided by the RIKEN Bio Resource Center, Japan, through the National Bio-Resource Project of the Ministry of Education, Culture, Sports, Science and Technology, Japan [38]. IFNAR^-/-^ (NIID) mice were obtained from the latter by mating with C57BL/6J mice, crossing F1 × F1 double heterozygote offspring to generate F2 IFNAR^-/-^/DNase2a^+/+^ mice, mating F2 × F2 IFNAR^-/-^/DNase2a^+/+^ mice to generate F3 offspring, from which the IFNAR^-/-^ (NIID) mouse line was established. IFNAR^-/-^ mice were inoculated subcutaneously (s.c.) or i.c. with the indicated ZIKV doses. Female IFNAR^-/-^ mice (> 7 weeks old) were paired with C57BL/6J (> 8 weeks old) purchased from SLC Ltd. (Shizuoka, Japan) as described previously [40]. When a plug was detected, this was deemed embryonic day 0.5 (E0.5). Weight gain was used to confirm pregnancy. On the indicated days, dams were infected s.c. with 1 × 10^4^ PFU of MR766, PRVABC59 or MR/PR(prM). The dams were euthanized and the indicated tissues were harvested at 3 dpi. Neonatal C57BL/6J mice were purchased from SLC Ltd. and inoculated i.c. with 1 × 10^4^ PFU of MR766 or PRVABC59.

### *In vivo* blood-brain barrier (BBB) permeability assay

BBB permeability was assessed with sodium fluorescein salt (NaF; 376 Da, Sigma Aldrich, Tokyo, Japan). Mice were injected intraperitoneally with 10 mg of NaF in 100 μl sterile saline. After 30 min to allow circulation of the NaF, peripheral blood was collected from the tail vein. Mice were sacrificed and perfused with 50 ml of cold PBS through the left ventricle of the heart to flush out intravascular fluorescein. Serum was diluted 1:10 in 20% trichloroacetic acid. The brain tissues were homogenized in PBS and diluted 1:10 in 20% trichloroacetic acid. Diluted serum and homogenized tissue samples were incubated in trichloroacetic acid at 4°C for 24 hrs. Samples were centrifuged for 15 min at 10,000 × *g* at 4°C to remove insoluble precipitates. After the addition of an equal volume of 75 mM borate buffer to the supernatant, the fluorescence intensity was determined by using a Synergy H4 Multi Mode Plate Reader with excitation at 460 nm and emission at 515 nm and Gen5 software (BioTek Instruments, Inc., Winooski, VT). A standard curve for quantitation of NaF in the samples was generated by analyzing samples of known NaF concentration in trichloroacetic acid and borate buffer in parallel. The degree of BBB permeability was measured as percentage (w/v) of NaF in a gram of brain tissue per the amount of NaF in a microliter of serum.

### Histology and immunohistochemistry

Tissue samples were fixed in 10% phosphate-buffered formalin, embedded in paraffin, sectioned, and stained with hematoxylin and eosin (H&E). Immunohistochemistry (IHC) was performed using an anti-ZIKV NS1 antibody (C01886G, Meridian Bioscience, Cincinnati, OH) as the primary antibody [41]. Specific antigen-antibody reactions were visualized by 3,3-diaminobenzidine tetrahydrochloride staining using a VECTASTAIN ABC HRP system (Vector Laboratories, Burlingame, CA).

### Virus titration

Serum was obtained from blood from tail vein bleeds and stored at -80°C. The indicated tissues were collected at the indicated time points and homogenized in Eagle’s minimal essential medium (Sigma Aldrich) containing 2% fetal bovine serum (FBS) using a tissue homogenizer and zirconia beads. The 50% cell culture infective dose (CCID_50_) assays for serum and supernatants from homogenized tissues were performed as described previously [41–50]. Briefly, serum or supernatants from tissues were titrated in duplicate or quadruplicate in 5- or 10-fold serial dilutions on C6/36 cells. After 5 days, 25 μl of supernatants was individually transferred onto parallel plates (i.e., A1 to A1, A2 to A2… H12 to H12) containing Vero cells. After another 5–7 days, cytopathic effects were confirmed under a microscope. The titer was calculated by the method of Reed and Muench [51].

### Reverse transcription, quantitative PCR (qRT-PCR)

qRT-PCR was performed as previously described [44]. Briefly, tissues were placed into RNAlater (Ambion, Austin, TX), and RNA was extracted with TRIzol (Life Technologies, Carlsbad, CA) from homogenized tissues prepared with ceramic beads and homogenizer according to the manufacturer’s instruction. For cultured cells, TRIzol was added directly to the cells and RNA was purified. cDNA was generated using an iScript cDNA Synthesis Kit (Bio-Rad, Hercules, CA). qPCR was performed using iTaq Universal SYBR Green Supermix (Bio-Rad) with the primers described in S1 Table. Values were normalized using an internal control (house keeping) gene, mouse RPL13A [52].

### *In vitro* transcytosis assay

bEnd.3 cells were seeded onto the luminal side of the Transwell filter (0.4 μm pore size, 6.5 mm diameter; Corning, NY). After the bEnd.3 monolayers had grown to confluency, each of MR766, PRVABC59 and MR/PR(prM) was added to the top well at a multiplicity of infection (MOI) of 1 per cell. The medium in the top and bottom wells was collected, and viral titers were determined by CCID_50_ assays as described above.

To assess permeability, NaF was added to the top wells and PBS to the bottom wells. After 30 min, samples from the bottom wells were analyzed for NaF concentration using a Synergy H4 Multi Mode Plate Reader with excitation at 460 nm and emission at 515 nm and Gen5 software. The ratio (%) of virus-induced permeability was calculated relative to a linear standard curve where “no virus control” was 0% and “no cell control” was 100%.

To measure viral uptake by bEnd.3 cells, the cell monolayers were infected at a MOI of 1 for each ZIKV. At the indicated time points, bEnd.3 cells were washed three times with PBS, trypsinized, washed three times with PBS, and dissolved in TRIzol. The extracted RNA was then subjected to qRT-PCR to measure the ZIKV genome level as described above.

### Western blot analyses

The culture medium of Vero E6 cells infected with each ZIKV were overlaid on 20% sucrose and ultracentrifuged at 28,000 × *g* at 4°C for 2 hrs, and viruses were recovered from the pellet. The proteins were separated by sodium dodecyl sulphate polyacrylamide gel electrophoresis (SDS-PAGE) with 15% polyacrylamide gels at 100 V for 2 hrs and transferred onto Immobilon-P membrane (Millipore Corp, Bedford, MA) at 100 V for 1 hr. Membranes were blocked with 3% skim milk in PBS for 2 hrs at room temperature followed by the addition of diluted hyperimmune mouse sera against ZIKV. The membranes were incubated with horseradish peroxidase-conjugated anti-mouse IgG antibody (Cell Signaling Technology, Danvers, MA). To produce hyperimmune sera against ZIKV, IFNAR^-/-^ mice were initially infected s.c. with 1 × 10^4^ CCID_50_ of Natal RGN strain (GenBank accession number: KU527068), and 3 weeks later, the mice were infected s.c. with 1 × 10^3^ CCID_50_ of MR766 twice more separated by a 3-week interval. The serum samples were collected 4 days after the second MR766 infection. The bound antibodies onto the membrane were visualized with chemiluminescence (Clarity Western ECL Substrate, Bio-Rad). Band densities were determined by using ImageJ software (US National Institutes of Health, Bethesda, MA).

### pH stability assay

To analyze the pH stability of viruses, a pH stability assay was performed as previously described [53], with modifications. Briefly, seven different sets of citrate phosphate buffers were prepared in the range between pH 5.0 and 8.0 according to McIlvaine’s standards [54]. A 1.5 × 10^3^ CCID_50_ aliquot of each virus was subjected to pH gradient treatment in the range between pH 5.0 and 8.0 for 30 min at room temperature and stored at -80°C until analysis. The virus titer in each sample was determined by CCID_50_ assays on Vero E6 cells as described above. Relative infectivity was determined by calculating the ratio of the number of CCID_50_ in pH-treated versus the respective non-treated viruses controls (kept at room temperature for 30 min).

### Molecular modeling and simulation

The Molecular Operating Environment (MOE) 2019.01 (Chemical Computing Group, Inc., Montreal, Quebec, Canada) was used for molecular computational chemistry. Homology modeling, energy minimization and protein surface patch analysis were conducted in the setting of the Amber10:EHT force field and the generalized Born/volume integral (GB/VI) implicit solvent model. The Protein Data Bank (PDB) deposited structures of ZIKV (PDB accession codes: 5U4W and 5IZ7), dengue virus (PDB accession code: 4B03) and West Nile virus (WNV, PDB accession code: 3C6E) were used as the templates. The default MOE settings were used for all parameters.

### Primary cells from IFNAR^-/-^ mice

The mouse embryonic fibroblasts (MEFs) were prepared as previously described [55], with modifications. Briefly, the MEFs were obtained from the IFNAR^-/-^ mouse embryos (day 16 of gestation) and cultured in Dulbecco’s Modified Eagle’s Medium (Sigma Aldrich) supplemented with 10% FBS, L-glutamine (Thermo Fisher Scientific), 2-mercaptoethanol (Thermo Fisher Scientific) and penicillin-streptomycin (Nacalai Tesque, Tokyo, Japan). After 4 days the cells were seeded in 24-well plates at a density of 1 × 10^5^ /cm^2^, incubated overnight and inoculated with each virus at a MOI of 0.1 or 1.

The neurons were isolated and cultured from the IFNAR^-/-^ mouse embryos (day 17 of gestation) using the Pierce Primary Neuron Isolation Kit (Thermo Fisher Scientific) following the manufacturer’s instructions. After 7 days, the cells were inoculated with each virus at a MOI of 0.1.

The bone marrow derived macrophages were prepared as previously described [56], with modifications. Briefly, bone marrow was collected from femurs of 13–20-week-old IFNAR^-/-^ mice. Cells were seeded in Dulbecco’s Modified Eagle’s Medium (Sigma Aldrich) supplemented with 10% FBS, penicillin-streptomycin (Nacalai Tesque) and 50 ng/ml M-CSF (PeproTech, Inc., Rocky Hill, NJ). The cell culture medium was changed every 2–3 days. After 5 days, the cells were seeded in 24-well plates at a density of 1 × 10^5^ /cm^2^, incubated overnight and inoculated with each virus at a MOI of 0.1. The MEFs, neurons and macrophages were cultured at 37°C in 5% CO_2_ incubators.

### Statistical analyses

The Student *t*-test was performed for normally distributed data sets where differences in variance were <4, skewness was >-2 and kurtosis was <2. The Kolmogorov-Smirnov test was used for non-parametric data where differences in variance were >4, skewness was <-2 and kurtosis was >2. The log rank test was used for statistical analysis of survival rates. Repeated-measures ANOVA was used to determine differences in viremia levels over time. Pearson or Spearman correlation analysis was performed for normal distributed data or for non-parametric data, respectively. A *p* value <0.05 was considered to indicate statistical significance. Statistical analysis of experimental data was performed using IBM SPSS Statistics for Windows, Version 22.0 (IBM Corp., Armonk, NY) or JMP 13 software (SAS Institute, Inc., Cary, NC).

## Results

### Virulence of MR766 and PRVABC59 in adult IFNAR^-/-^ mice

Although significant differences in virulence between MR766 and PRVABC59 strains of ZIKV in IFNAR^-/-^ mice after s.c. infection have been reported previously (MR766 and PRVABC59 caused 100% and 0% mortality, respectively) [19], we repeated this comparison herein as both the IFNAR^-/-^ mice and the MR766 strain available at NIID were distinct (see Materials and Methods). The MR766-NIID strain (hereafter referred to as MR766) does not have the consensus sequence for N-linked-glycosylation in the E protein (S1A Fig) [34] (GenBank accession number: LC002520). N-linked-glycosylation was previously reported to be associated with the virulence of ZIKV [57,58].

**Fig 1:**
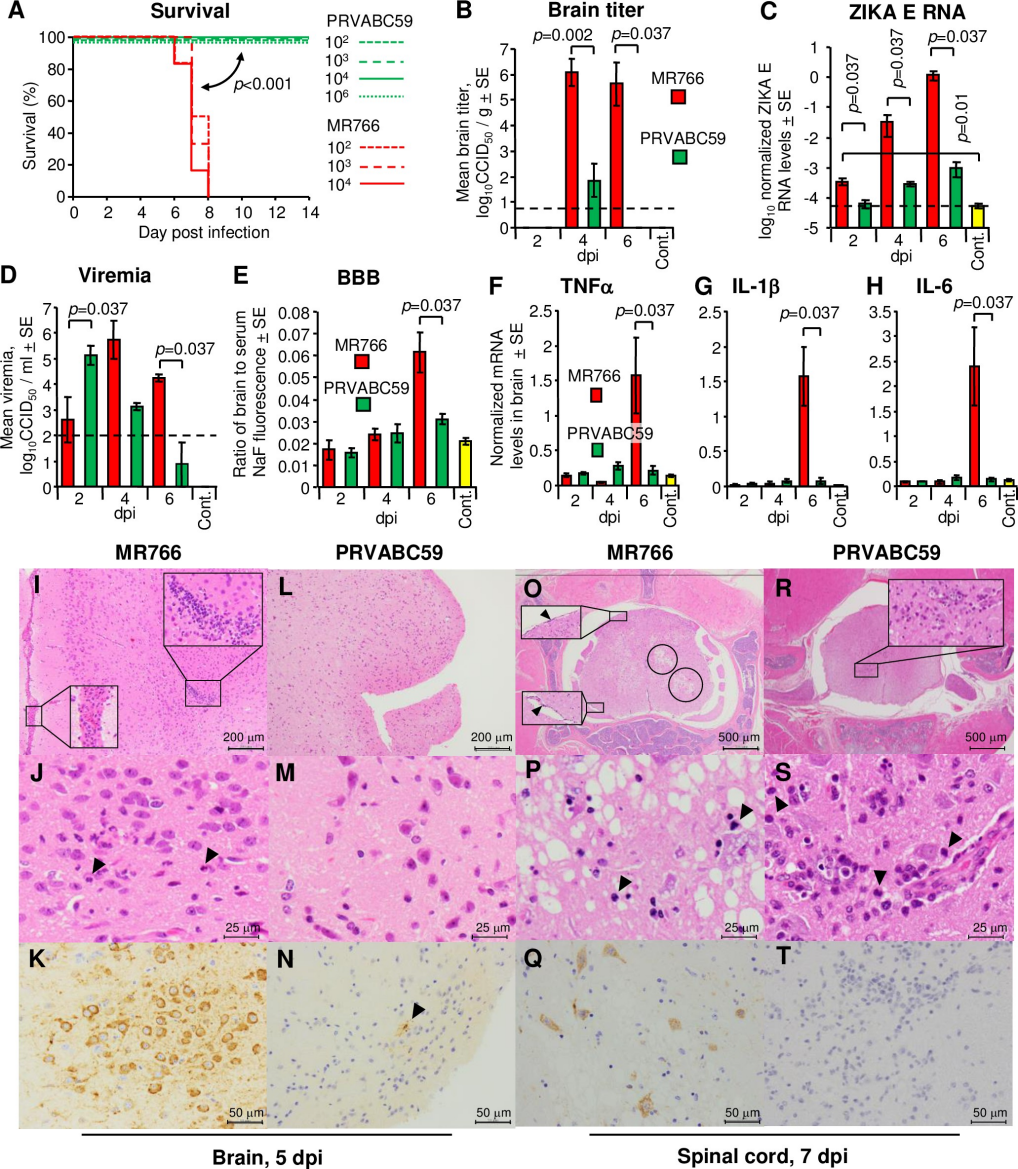
Virulence of MR766 and PRVABC59 after subcutaneous infection in IFNAR^-/-^ mice. (A) Survival of 7 to 32-week-old IFNAR^-/-^ mice after s.c. infection with MR766 or PRVABC59. Mice were infected with the indicated doses of MR766 or PRVABC59 and monitored until 14 dpi. Each group consisted of n = 6–12 mice, with similar numbers of male and female mice per group. Comparison of Kaplan-Meier survival curves between groups was performed by log-rank analysis. (B) Mean viral titers in the brain of mice s.c. infected with 1 × 10^3^ PFU of MR766 or PRVABC59 (n = 4 mice per group). Brains were harvested at 2, 4 and 6 dpi, with tissue titers determined by CCID_50_ assays. Four uninfected mouse brains were used as controls (Cont.). Kolmogorov-Smirnov test was used for statistical analysis. Limit of detection was 0.83 log_10_CCID_50_/g indicated by the horizontal dashed line. (C) ZIKV RNA levels in brains of mice s.c. infected with 1 × 10^3^ PFU of MR766 or PRVABC59 were determined by qRT-PCR with the E gene-specific primers (n = 4 mice per group). ZIKV RNA levels were normalized to RPL13 mRNA levels. Four uninfected mouse brains were used as controls (Cont.). Kolmogorov-Smirnov test was used for statistical analysis. Limit of detection is indicated by the horizontal dashed line. (D) Mean viremia titers of mice s.c. infected with 1 × 10^3^ PFU of MR766 or PRVABC59 (n = 4 mice per group). Sera were collected at 2, 4 and 6 dpi, and titers were determined by CCID_50_ assays. Four uninfected mouse sera were used as controls (Cont.). Kolmogorov-Smirnov test was used for statistical analysis. Limit of detection was 2 log_10_CCID_50_/ml indicated by the horizontal dashed line. (E) Blood-brain barrier permeability. IFNAR^-/-^ mice were infected s.c. with 1 × 10^3^ PFU of MR766 or PRVABC59 (n = 4 mice per group). At 2, 4 and 6 dpi, mice received 10 mg sodium fluorescein intraperitoneally. Sera and brains were harvested after 30 min. The ratio of brain to serum fluorescence was determined for each animal, and the mean values are shown. Four uninfected mice were used as controls (Cont.). Kolmogorov-Smirnov test was used for statistical analysis. (F) TNFα mRNA levels in brains of mice infected s.c. with 1 × 10^3^ PFU of MR766 or PRVABC59 (n = 4 mice per group). Four uninfected mice were used as controls (Cont.). Cytokine mRNA levels were normalized to RPL13 mRNA levels. Kolmogorov-Smirnov test was used for statistical analysis. (G) As in (F) for IL-1β mRNA. (H) As in (F) for IL-6 mRNA. (I) H&E staining of meninx and brain parenchyma at 5 dpi with MR766. Inserts show cellular infiltrates. (J) As in (I) with higher magnification showing neuronophagia (arrowheads). (K) IHC of brain parenchyma at 5 dpi with MR766 using anti-ZIKV NS1 antibody. Positive signal (brown) was seen in cells with neuronal morphology. (L-N) As in (I-K), after infection with PRVABC59. (O) H&E staining of spinal cord at 7 dpi with MR766. Black circles indicate vacuolation of the parenchyma. Inserts with arrowheads show infiltrates in the meninx. (P) As in (O) with higher magnification. Arrowheads indicate infiltration of neutrophils. (Q) IHC of spinal cord at 7 dpi with MR766 using anti-ZIKV NS1 antibody showing positive staining of neural cells. (R-T) As in (O-Q) after infection with PRVABC59.

IFNAR^-/-^ mice were infected s.c. with 1 × 10^2^, 1 × 10^3^ or 1 × 10^4^ PFU of MR766 or 1 × 10^2^, 1 × 10^3^, 1 × 10^4^ or 1 × 10^6^ PFU of PRVABC59. MR766-infected mice showed overt symptoms (weight loss, ruffled fur, hunched back, shivering, hypoactivity and/or leg paralysis) and reached defined endpoints for euthanasia between 6 and 8 dpi, irrespective of the inoculation dose, gender or age (7–32 weeks, Figs 1A, S2A and S2B). In contrast, PRVABC59-infected mice showed no overt symptoms, and all survived, irrespective of the inoculation dose, gender or age (Figs 1A, S2A and S2B). Survival rates of MR766-infected mice were significantly lower than those of PRVABC59-infected mice for all comparisons (e.g., survival of 1 × 10^2^ PFU of MR766-infected mice was lower than that of 1 × 10^2^, 1 × 10^3^, 1 × 10^4^ or 1 × 10^6^ PFU of PRVABC59-infected mice) (*p*<0.001, log rank statistic). These data suggested that the virulence of MR766 thus does not appear to depend on N-linked-glycosylation (S1 Fig).

Higher morbidity and mortality in mice s.c. infected with MR766 was associated with higher levels of ZIKV in the brain compared with those in PRVABC59-infected mice (Fig 1B and 1C). The viral titers in the brain of MR766-infected mice were significantly higher than those of PRVABC59-infected mice at 4 and 6 dpi (Fig 1B). The viral RNA levels in the brain of MR766-infected mice were significantly higher than those in PRVABC59-infected mice at 2, 4 and 6 dpi (Fig 1C), with the viral titer data. The mean viremia levels in MR766-infected mice were lower or similar when compared to those in PRVABC59-infected mice at 2 and 4 dpi (Fig 1D), arguing that the higher levels of MR766 in the brain at 2 and 4 dpi were not associated with increased viremia levels. Furthermore, the viremia levels, which are shown in S2D Fig, were not good predictors of survival. For instance, at a s.c. inoculation dose of 1 × 10^4^ PFU, mean viremia levels in MR766- and PRVABC59-infected mice were not significantly different (*p* = 0.91), yet survival rates were 0% and 100%, respectively (Fig 1A). The mean viremia levels for s.c. inoculations of 1 × 10^2^ PFU of MR766 and 1 × 10^4^ PFU of PRVABC59, 1 × 10^3^ PFU of MR766 and 1 × 10^4^ PFU of PRVABC59, 1 × 10^3^ PFU of MR766 and 1 × 10^6^ PFU of PRVABC59, or 1 × 10^4^ PFU of MR766 and 1 × 10^6^ PFU of PRVABC59 were also not significantly different (*p* = 0.32, *p* = 1.00, *p* = 0.35 or *p* = 0.06, respectively, S2D Fig), but survival rates of MR766- and PRVABC59-infected mice were again 0% and 100%, respectively (Fig 1A).

**Fig 2:**
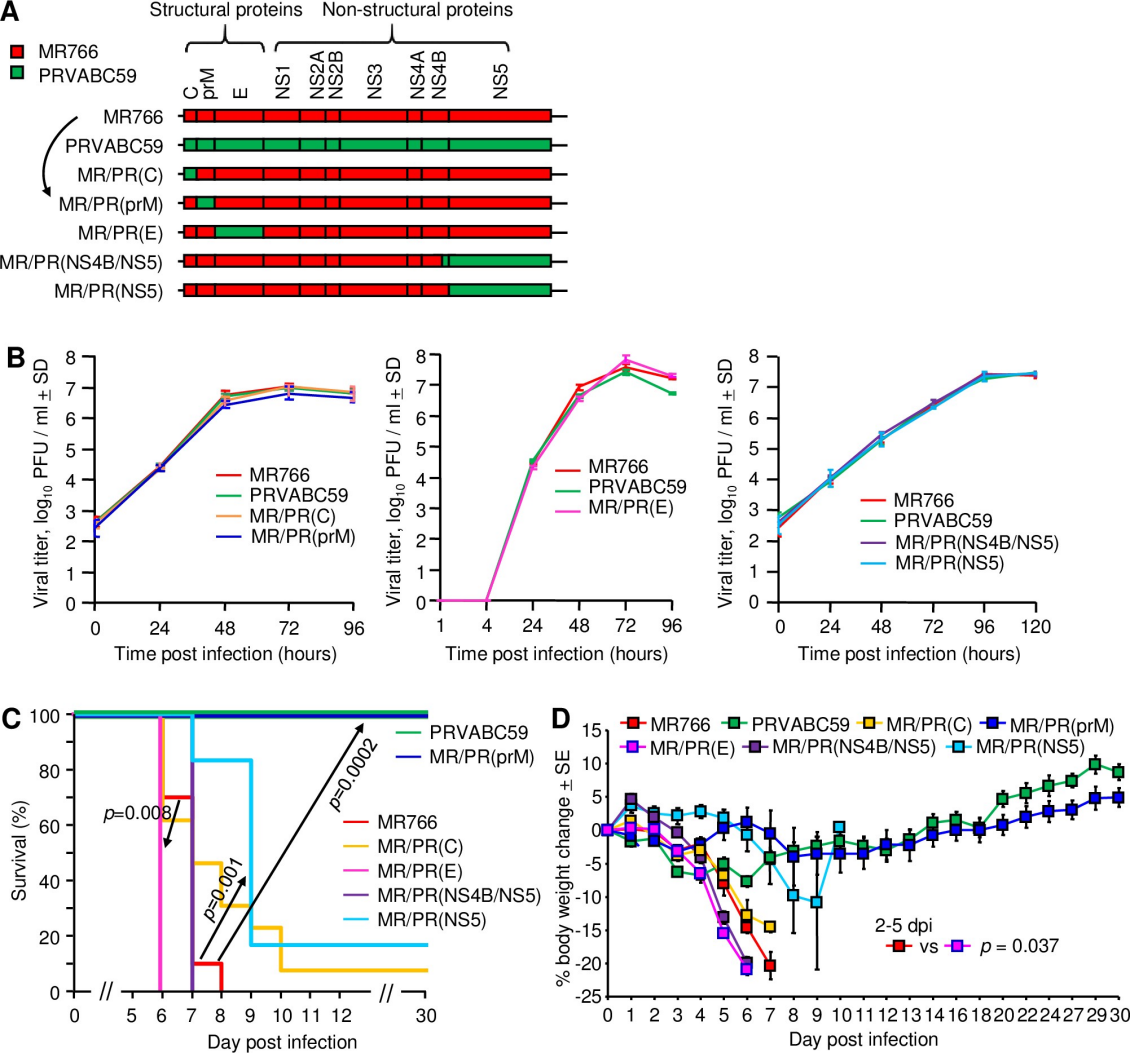
Chimeric MR766/PRVABC59 viruses and their virulence in IFNAR^-/-^ mice. (A) Schematic representation of viral genes of wild-type and chimeric ZIKVs. (B) Growth kinetics of wild-type and chimeric viruses *in vitro*. Vero cells were infected at a MOI of 0.01, and the supernatant was collected at the indicated times. The viral titres were determined by plaque assay on Vero cells [34]. Each data point represents the average of 3 or 4 wells. (C) Survival of 8–14-week-old IFNAR^-/-^ mice s.c. infected with 1 × 10^4^ PFU of MR766 (n = 10), PRVABC59 (n = 12) or each chimeric virus (n = 6–13). Comparisons of Kaplan-Meier survival curves between different groups were performed by log-rank analysis. The indicated comparisons are between MR766 vs. MR/PR(prM) (*p* = 0.0002), MR766 vs. MR/PR(NS5) (*p* = 0.001) and MR766 vs. MR/PR(E) (*p* = 0.008). (D) Mean percent weight change relative to day 0 after s.c. infection with 1 × 10^4^ PFU of MR766 (n = 12), PRVABC59 (n = 6) or each chimeric virus (n = 6). Seven to nine-week-old IFNAR^-/-^ mice were s.c. infected with 1 × 10^4^ PFU of each virus. Statistical analysis was performed by repeat measure ANOVA for 2 to 5 dpi.

The BBB was significantly more permeable in MR766-infected mice at 6 dpi, as measured by fluorescein entry into the brain (Fig 1E). TNFα, IL-6 and IL-1β were reported to be associated with disruption of the BBB [30,59,60]. Consistent with the latter publications, the brain mRNA levels of these cytokines were significantly higher only on 6 dpi in the MR766-infected mice (Fig 1F–1H). Serum levels of these cytokines were not significantly different (S2E Fig). However, ZIKV was reported to enter the brain in IFNAR^-/-^ mice via transcytosis through brain endothelial cells rather than by disruption of the BBB [61], with similar results reported for other flaviviruses [62–65]. Moreover, BBB permeability was shown not to be a primary determinant for neurotropic flavivirus lethality in rodents [66]. Higher levels of MR766 were seen in the brain 2–4 days before (Fig 1B, 4 dpi and Fig 1C, 2 and 4 dpi) increased BBB permeability was evident (Fig 1E, 6 dpi); our results are therefore consistent with the aforementioned publications. Taken together these observations argue that disruption of the BBB is not responsible for the increased virulence of MR766.

Subcutaneous infection of MR766 revealed inflammatory infiltrates in the meninx (Fig 1I, bottom left insert) and parenchyma (Fig 1I, top right insert) of brain tissues. Neuronophagia was also evident (Fig 1J, arrowheads). The presence of virus-infected cells with neuronal morphology (Fig 1K, dark brown staining) confirmed the tropism of ZIKV as previously published [67]. Infiltrates and viral protein were rare (only one NS1-positive cell in 3 mouse brains; Fig 1N, arrowhead) in brain sections of PRVABC59-infected mice (Fig 1L–1N). The spinal cord of MR766-infected mice showed parenchymal vacuolation, which indicates neural tissue degeneration (Fig 1O, black circles). Meningeal inflammation was also observed (Fig 1O, inserts, arrowheads). At higher magnification, H&E staining suggested neuronophagia and neuronal loss with infiltration of neutrophils (arrowheads) (Fig 1P). IHC with anti-NS1 showed positive signals in cells with neuronal morphology (Fig 1Q, dark brown staining). The spinal cord of PRVABC59-infected mice showed focal inflammatory infiltrates (Fig 1R, insert; Fig 1S, arrowheads), whereas no significant anti-NS1 staining was evident (Fig 1T). H&E and IHC of spleen, liver and kidney of the infected mice showed no notable features. These H&E and IHC results are thus consistent with the higher brain viral titers for MR766-infected mice (Fig 1B).

### Virulence of chimeric MR766/PRVABC59 viruses in IFNAR^-/-^ mice

To identify the proteins that might be responsible for enhanced virulence of MR766, five MR766-PRVABC59 chimeric viruses were constructed using an infectious molecular clone of MR766 [34]. The five MR766 chimeras contained C, prM, E, NS4B/NS5 or NS5 of PRVABC59 (Fig 2A). These proteins were chosen because the structural proteins (C-prM-E) of African and Asian ZIKVs have been reported to differ in their ability to infect human neuronal cells [68]. The C-terminal of flavivirus NS4B protein was reported to be critical for mouse cell adaptation and neurovirulence in mice [69,70]. Finally NS5, encoding both the viral methyltransferase and RNA-dependent RNA polymerase, was shown to inhibit type I and III IFN responses via IRF3 antagonism or STAT2 degradation [71–74]. The *in vitro* growth kinetics of all five chimeras and the parental viruses were similar, illustrating that the gene swapping had no discernable effect on replication competence (Fig 2B).

Adult IFNAR^-/-^ mice were challenged s.c. with 1 × 10^4^ PFU of each of the viruses. Infection with MR766 resulted in 100% mortality, whereas infection with PRVABC59 resulted in 100% survival (Fig 2C), as described above (Fig 1A). Replacing the prM of MR766 with that of PRVABC59 (Fig 2A, black arrow) conferred significantly reduced virulence with 100% of mice infected with the prM chimera surviving (Fig 2C, MR766 vs. MR/PR(prM), black arrow, *p* = 0.0002). Two of 12 (16.7%) MR/PR(prM)-infected mice showed a self-resolving, mild hind-leg paralysis, whereas all MR766-infected mice showed overt terminal neurological symptoms. All other chimeras showed >80% mortality (Fig 2C) and significant reductions in body weight (Fig 2D). The prM of MR766 thus appeared to be the major determinant of MR766’s virulence after s.c. infection in adult IFNAR^-/-^ mice. NS5 also showed a relatively lower effect on virulence than prM, with slightly, but significantly longer survival seen after MR/PR(NS5) infection (Fig 2C, *p* = 0.001).

To confirm these results, a repeat experiment was conducted, and mice were infected s.c. with newly prepared viral stocks for MR766, PRVABC59 and MR/PR(prM). The viral genome sequences of newly prepared MR766, PRVABC59 or MR/PR(prM) stocks were confirmed to be identical to GenBank LC002520, KU501215 or LC571075, respectively. Infection of adult IFNAR^-/-^ mice with MR766 again resulted in 100% mortality, whereas infection with PRVABC59 or MR/PR(prM) resulted in 100% survival (S3A Fig). No additional mutations/substitutions compared to viral stock (GenBank accession number: LC571075) were found in prM when MR/PR(prM) was recovered from infected mouse sera at 4 dpi (n = 2).

Although the viremia levels of the 7 different viruses varied by 2–3 logs (S3B Fig), neither mean viremia levels for 1–6 dpi (S3C Fig), mean viremia level at 6 dpi (S3D Fig), nor peak viremia levels (S3E Fig) correlated significantly with survival (consistent with the observations in S2D Fig).

As the results above illustrated that prM of MR766 represents a virulence determinant for MR766, two more chimeras were constructed, MR/PR(pr) and MR/PR(M), wherein the MR766 genes coding for pr or M where replaced with corresponding genes from PRVABC59 (S4A Fig). The growth kinetics of the chimeric and the parental viruses *in vitro* were similar (S4B Fig). Adult IFNAR^-/-^ mice were challenged s.c. with 1 × 10^4^ PFU of each of the viruses. Infection with MR766 resulted in 100% mortality, whereas infection with PRVABC59 resulted in 100% survival (S4C Fig), as described above (Figs 1A, 2C and S3A). After s.c. infection with 1 × 10^4^ PFU of MR/PR(M), 83.3% of the infected mice succumbed to the infection, indicating that virulence was only slightly and not significantly reduced. Replacing the pr of MR766 with that of PRVABC59 conferred significantly reduced virulence with 33.3% survival (S4C Fig, MR766 vs. MR/PR(pr), black arrow, *p* = 0.039); however, virulence was not as dramatically reduced as was seen after MR/PR(prM) infection (100% survival, Figs 2C and S3A). These observations indicated that amino acids in both MR766’s pr and M proteins synergistically contributed to the virulence imparted by MR766’s prM protein.

### Role of prM cleavage or pH stability in virulence

**Fig 3:**
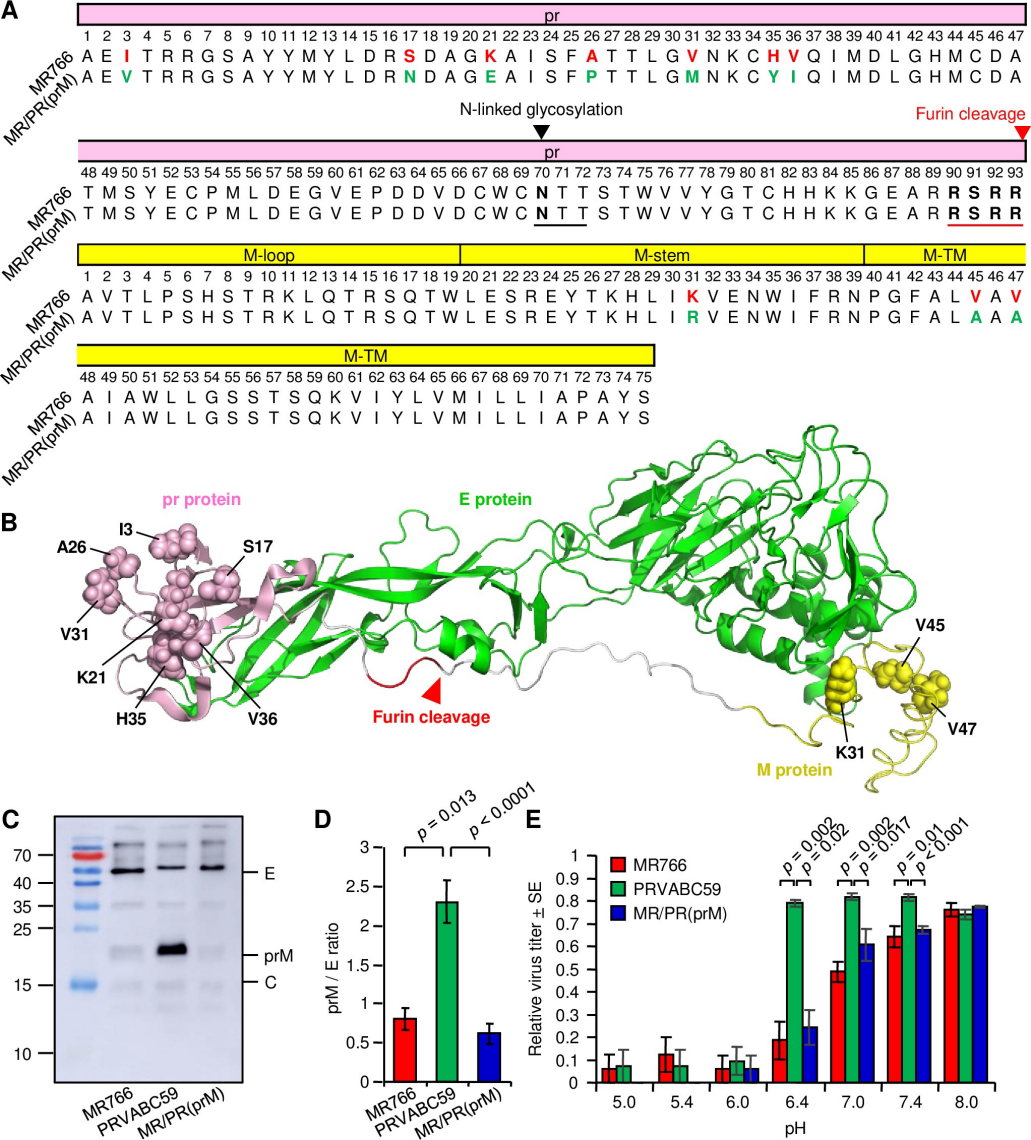
prM cleavage and pH stability. (A) Sequence alignment of prM from MR766 and MR/PR(prM) (or PRVABC59). The N-linked-glycosylation site (black arrowhead) and motif (underlined) are shown. The furin cleavage site (red arrowhead) and motif (red underline) are indicated. (B) Structure prediction of MR766 prME monomer in the immature virus. The homology model based on dengue virus structure (PDB accession code: 4B03) was generated by MOE homology modeler. The pr, M and E proteins are shown in light pink, yellow and green, respectively. Different amino acid residues in prM protein between MR766 and MR/PR(prM) are shown as atomic spheres. The furin cleavage site (red arrowhead) and motif (red) are indicated. (C) Representative example of a Western blot of purified MR766, PRVABC59 and MR/PR(prM). (D) Densitometry analyses of prM and E proteins from Western blots of purified MR766, PRVABC59 and MR/PR(prM). The intensities of the prM and E bands were determined using ImageJ software, with the mean (7 purified preparations of MR766, 6 purified preparations of PRVABC59 and MR/PR(prM)) prM/E ratio shown. Statistical analysis was performed by *t*-test. (E) pH stability of MR766, PRVABC59 and MR/PR(prM). MR766 (n = 6 replicates), PRVABC59 (n = 8) and MR/PR(prM) (n = 6) were treated at the indicated pH for 30 min, and virus titers were determined by CCID_50_ assays. Kolmogorov-Smirnov test or *t*-test was used for statistical analysis.

Increased levels of cleavage of prM (to pr and M) by furin have been reported to increase flavivirus virulence [75–77]. Thus, the reduced virulence of MR/PR(prM) compared to MR766 might be due to reduced prM cleavage. Alignment of the prM sequences of MR766 and MR/PR(prM) (or PRVABC59) illustrated that amino acid differences (red to green) were not located near the furin cleavage site (Fig 3A). Modeling also confirmed that these amino acids (shown as atomic spheres) were not located near the furin cleavage site in the tertiary structure (Fig 3B) and therefore were unlikely to affect furin cleavage.

To provide experimental evidence for prM cleavage, purified preparations of MR766, PRVABC59 and MR/PR(prM) were analyzed by Western blotting with mouse polyclonal convalescent ZIKV antisera (Fig 3C). As documented previously for dengue virus, M protein was not detectable in such immunoblot analyses [6,78], and there were no antibodies available that react with pr or M. By using densitometry of Western blots, the ratios of prM (uncleaved) over E were calculated for 6–7 purified preparations of MR766, PRVABC59 and MR/PR(prM) in repeated experiments (representative example shown in Fig 3C). The mean prM/E ratios were not significantly different between MR766 and MR/PR(prM) (Fig 3D). These experiments did not support the contention that reduced prM cleavage was responsible for the reduced virulence of MR/PR(prM). The prM/E ratio for PRVABC59 was significantly higher than that for MR766 (Fig 3D), perhaps consistent with the lower virulence seen in Fig 1A. During the viral maturation process, the trimeric prM–E spikes on the immature virion rearranged into prM–E dimers that lie flat on the virus surface [79]. This structural rearrangement facilitates furin cleavage of pr from the prM, with the E protein playing a significant role in facilitating the structural change [80]. There are 17 amino acid differences between the E protein of PRVABC59 and MR766 (and MR/PR(prM)), with one or more of these differences perhaps responsible; mutations in E have previously been shown to affect prM cleavage [80,81].

After prM cleavage in the infected cell, the pr protein remains attached to the virion to prevent premature fusion in the low pH environment of the Golgi. After release from the cell, pr dissociates from E in the extracellular neutral pH environment. To infect a new cell, the virus enters via an endosome, and fusion is triggered by a reduction in the endosomal pH [53,82]. Infectious viruses treated at low pH *in vitro* undergo premature fusion and lose their infectivity (Fig 3E). MR766 and MR/PR(prM) showed similar pH stability, with indistinguishable pH versus infectivity profiles (Fig 3E). The 3 amino acid changes in M would thus appear not to influence E–M interactions [83] sufficiently to affect pH stability. In addition, pH stability would not appear to be a determining factor in the reduced virulence of MR/PR(prM) when compared with MR766. PRVABC59 virions showed significantly higher pH stability, again a feature likely associated with the amino acid differences in the E proteins [84]. The amino acids located at the interface between domain I and II of the E protein influence the pH threshold for fusion [85]. There were two amino acid differences between MR766 and PRVABC59 (at positions 283 and 285) that are located in this interface area, and may thus be responsible for the pH stability differences between PRVABC59 and MR766 (and (MR/PR(prM)).

### Acquisition of N-linked glycosylation and survival

N-linked-glycosylation at 154 in the E protein was reported to be associated with the virulence of ZIKV [57,58]. The viral stocks of MR766, MR/PR(prM) and MR/PR(C) do not have the glycosylation motif, which comprises amino acids positions 154–156 (S5A Fig). To see whether the N-linked-glycosylation motif at 154 was restored *in vivo*, the viral sequences of MR766, MR/PR(prM) and MR/PR(C) recovered from infected mouse sera at 4 dpi was determined. In all mice infected with MR766, the glycosylation motif was restored by 4 dpi and all mice reached the ethical endpoint for euthanasia by 7/8 dpi (S5B Fig). The glycosylation motif was restored in half the mice infected with MR/PR(prM), yet all mice survived (whether the glycosylation motif had been restored or not). Similarly, the glycosylation motif was restored in half the mice infected with MR/PR(C), yet all mice died (S5B Fig). Thus, consistent with the data in (S1 Fig), in this context, acquisition of glycosylation at N154 did not correlate with survival.

### Role of prM protein of MR766 on neurovirulence and neuroinvasiveness

**Fig 4:**
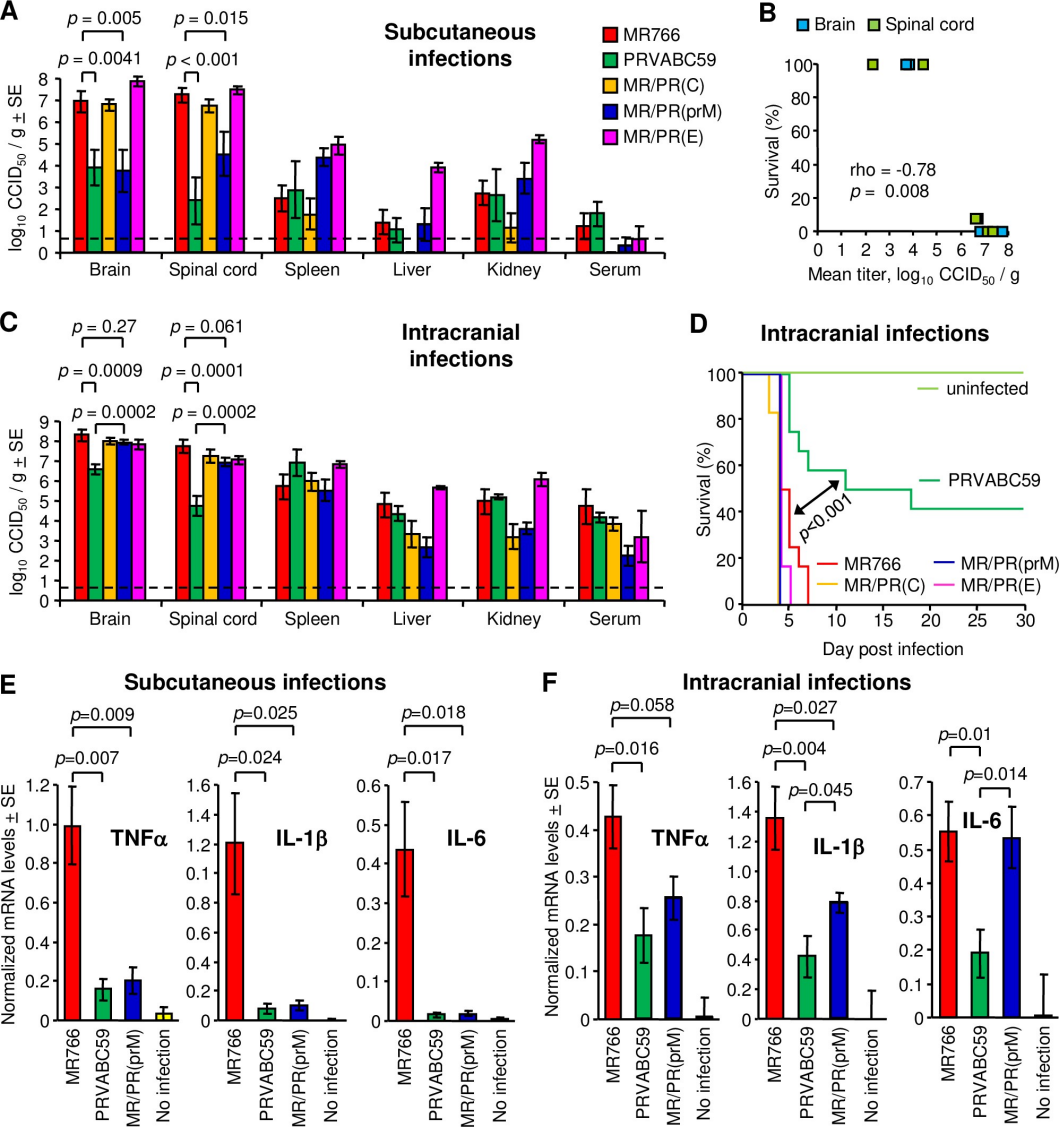
Viral titers in tissues and brain cytokine levels in IFNAR^-/-^ mice infected with wild-type or chimeric viruses. (A) Virus titers in tissues after s.c. infections with 1 × 10^4^ PFU of MR766, PRVABC59, MR/PR(C), MR/PR(prM) or MR/PR(E) (n = 5–10 mice per group). Organs were harvested at 6 dpi, and viral titers were determined by CCID_50_ assays. Limit of detection was 0.83 log_10_CCID_50_/g indicated by the horizontal dashed line. Kolmogorov-Smirnov test or *t*-test were used for statistical analyses. (B) Inverse correlation between percent survival and mean virus titer in the brain and spinal cord. Significance was determined by Spearman’s correlation test. (C) Virus titers in tissues after i.c. infections with 1 × 10^4^ PFU of MR766, PRVABC59, MR/PR(C), MR/PR(prM) or MR/PR(E) (n = 5–12 mice per group). Organs were harvested at 4 dpi, and viral titers were determined by CCID_50_ assays. Limit of detection was 0.83 log_10_CCID_50_/g indicated by the horizontal dashed line. Statistical analysis was performed by *t*-test. (D) Survival of 10–11-week-old IFNAR^-/-^ mice infected with 1 × 10^4^ PFU of MR766 (n = 12), PRVABC59 (n = 12), each chimeric virus (n = 6) or uninfected (n = 4). Comparisons of Kaplan-Meier survival curves between different groups were performed by log-rank analysis. (E) mRNA levels of TNFα, IL-1β and IL-6 in brains at 6 days after s.c. infections (n = 6 mice per group). Three uninfected mice were used as controls. Cytokine mRNA levels in brains were normalized to RPL13 mRNA levels. Statistical analysis was performed by *t*-test. (F) As in (E) at 4 days after i.c. infection.

To explore further the mechanism for the reduced virulence of MR/PR(prM) *in vivo* when compared to MR766, IFNAR^-/-^ mice were infected s.c. with 1 × 10^4^ PFU of MR766, PRVABC59, MR/PR(C), MR/PR(prM) or MR/PR(E), and tissue virus titers determined. The titers in the brain and spinal cord were significantly higher in MR766-, MR/PR(C)- and MR/PR(E)-infected mice than those in PRVABC59- and MR/PR(prM)-infected mice (Fig 4A). The titers in the brain and spinal cord also showed an inverse correlation with survival rates for these 5 viruses after s.c. infection (Fig 4B, rho = -0.78, *p* = 0.008). Correlations between survival rates and virus titers in the other tissues were not significant.

Adult IFNAR^-/-^ mice were challenged i.c. with 1 × 10^4^ PFU of MR766, PRVABC59, MR/PR(C), MR/PR(prM) or MR/PR(E). After s.c. infection, viral titers in the brain of MR766-infected mice were 3.13 logs higher than those of MR/PR(prM)-infected mice (*p* = 0.005, Fig 4A). However, after i.c. infection, the difference in virus titers in the brain between MR766- and MR/PR(prM)-infected mice was only 0.35 logs and did not reach statistical significance (*p* = 0.27, Fig 4C). The same contention was supported by the virus titers in the spinal cord of the mice infected with each of these two viruses (Fig 4A and 4C). These results argue that the primary mechanism for the difference between these MR766 and MR/PR(prM) was neuroinvasiveness rather than neurovirulence.

Intracranial infection with MR/PR(prM) resulted in 100% mortality, as was also the case for i.c. infection with MR766, MR/PR(C) and MR/PR(E) (Fig 4D). Thus, once MR/PR(prM) crossed into the brain, MR/PR(prM) had the same capacity as MR766 (and the other chimeras) to replicate sufficiently to cause a 100% lethal infection, yet s.c. MR/PR(prM) infection resulted in 100% survival (Fig 2C). These observations further support the aforementioned contention that the difference in virulence between MR766 and MR/PR(prM) was primarily associated with differences in neuroinvasiveness.

Intracranial infection with PRVABC59 resulted in significantly lower brain titers (Fig 4C) and significantly higher levels of survival (Fig 4D), when compared with MR766. MR766 is thus significantly more neurovirulent than PRVABC59 in adult IFNAR^-/-^ mice.

The cytokine levels in the brain after s.c. or i.c. virus inoculation (Fig 4E and 4F) largely paralleled the virus titers in the brain (Fig 4A and 4B) and thus did not provide insight regarding the reduced neuroinvasiveness of MR/PR(prM). Serum cytokine levels also did not provide insight into differences between MR766 and PRVABC59 (see S2E Fig). The S17N mutation in the pr protein was previously reported to be associated with increased microcephaly in wild-type neonatal mice after i.c. infection [16]. However, this observation was not supported by later studies [86,87]. Herein, the S17N substitution (Fig 3A) was not associated with significant differences in brain viral titers after i.c. infection (Fig 4C, *p* = 0.27).

### Fetal brain infections

**Fig 5:**
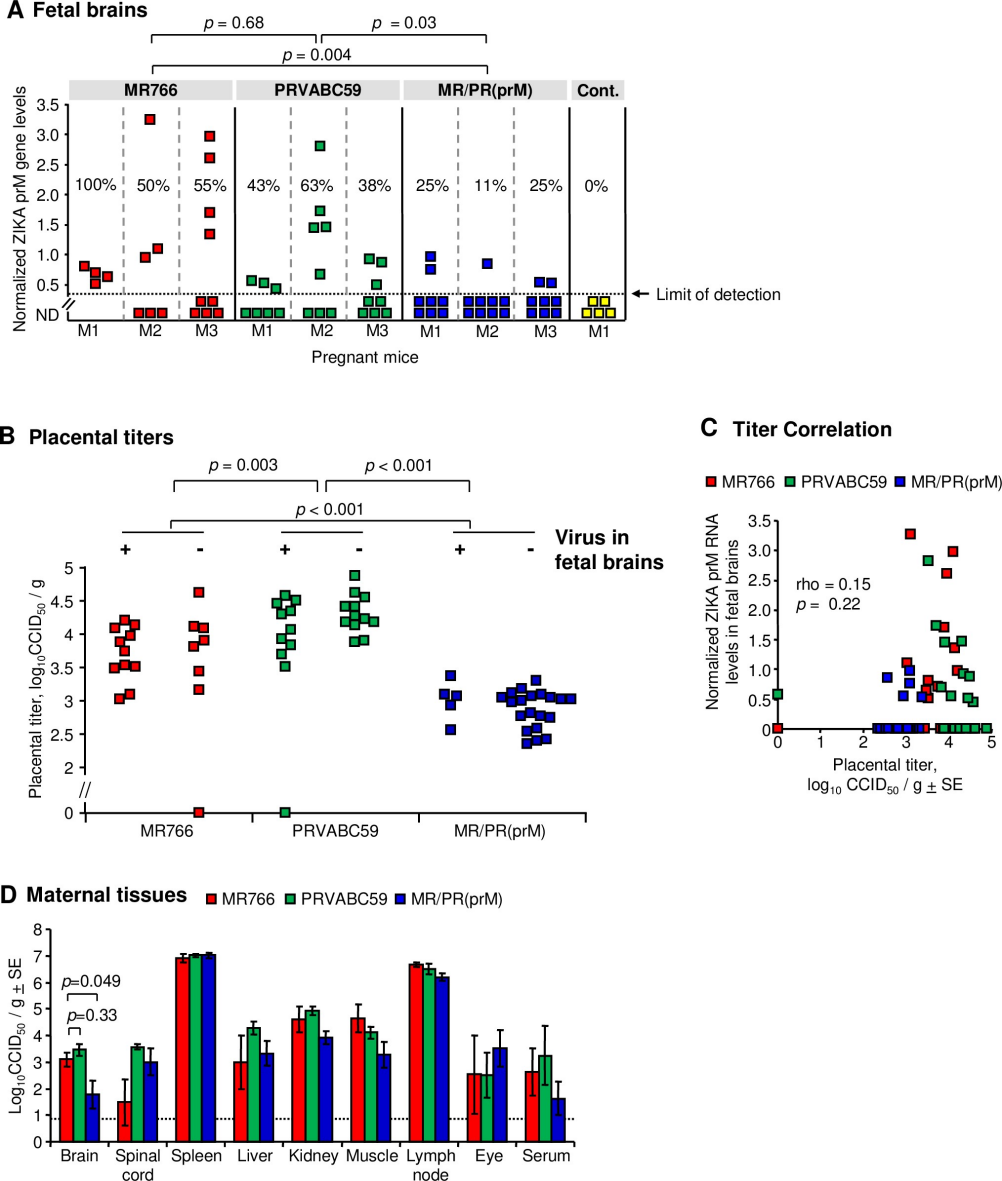
Fetal brain infections. (A) ZIKV RNA levels in fetal brains. Individual dams are indicated on the x axis; each square representing one fetus. Vertical dashed grey lines separate litters from each dam. qRT-PCR was performed using the prM gene-specific primers. ZIKV RNA levels were normalized to RPL13 mRNA levels. Five fetal brains from uninfected dam were used as controls. The percentage of fetuses that were infected for each dam at E18.5 are indicated (%). Statistics shown on the figure were performed by Kolmogorov-Smirnov tests. Limit of detection is indicated by the horizontal dashed line. ND, not detected. (B) Viral titers in the placentas. Viral titers were determined by CCID_50_ assays. + indicates the titers are from placentas of fetuses with detectable virus in the brain;—indicates the titers are from placentas of fetuses with no detectable virus in the brain. Kolmogorov-Smirnov test was used for statistical analysis. Limit of detection was 0.83 log_10_CCID_50_/g. (C) No correlation between placental viral titers and ZIKV RNA levels in fetal brains were found. Significance was determined by Spearman’s correlation test. (D) Viral titers in maternal tissues. Indicated tissues of pregnant mice were harvested at 3 dpi, and viral titers were determined by CCID_50_ assays (n = 4–8 mice per group). Limit of detection was 0.83 log_10_CCID_50_/g indicated by the horizontal dashed line. *T*-test was used for statistical analysis.

The mouse BBB becomes functional at E15.5 [88]. Pregnant IFNAR^-/-^ mice were infected s.c. with 1 × 10^4^ PFU of MR766, PRVABC59 or MR/PR(prM) at E15.5 and fetal brains, placenta and other tissues were harvested at E18.5. Both the viral RNA levels in the fetal brains (*p* = 0.004) and the percentage of fetuses infected at E18.5 (*p* = 0.046, 100, 50, 55 vs 25, 11, 25; Kruskal Wallis test) were significantly lower in fetuses from dams infected with MR/PR(prM) than those infected with MR766 (Fig 5A). Placental titers were significantly lower after infection with MR/PR(prM) (and slightly but significantly higher after PRVABC59 infection) (Fig 5B). However, whether fetal brain was infected or not, did not correlate with the corresponding placental titer for any of the 3 viruses (Fig 5B). Virus levels in the fetal brains and corresponding placentas also did not correlate (Fig 5C). Adult tissue titers for MR766 and MR/PR(prM) were not substantially or significantly different, except for brain (Fig 5D), consistent with 6 dpi data in Fig 4A.

Thus as for adult brains, infection of fetal brains was reduced after MR/PR(prM) infection, when compared with MR766. This data is consistent with the contention that MR/PR(prM) exhibits decreased neuroinvasiveness in both adults and fetuses. However, we cannot exclude the possibility that MR/PR(prM) has (or also has) a reduced capacity to cross the placenta and/or a reduced capacity to replicate in the fetal brain.

### Efficiency of MR/PR(prM) and MR766 in crossing the BBB via transcytosis

MR/PR(prM) showed a reduced ability to enter the brain compared with MR766 (Figs 4 and 5A). A standard *in vitro* method for evaluating virus infection-induced BBB permeability [89–91] was found to be unsuitable due to the significantly higher replication of MR766 (when compared with PRVABC59 and MR/PR(prM)) in the C8D1A astrocytoma cell line used in this assay (S7 Fig).

**Fig 6:**
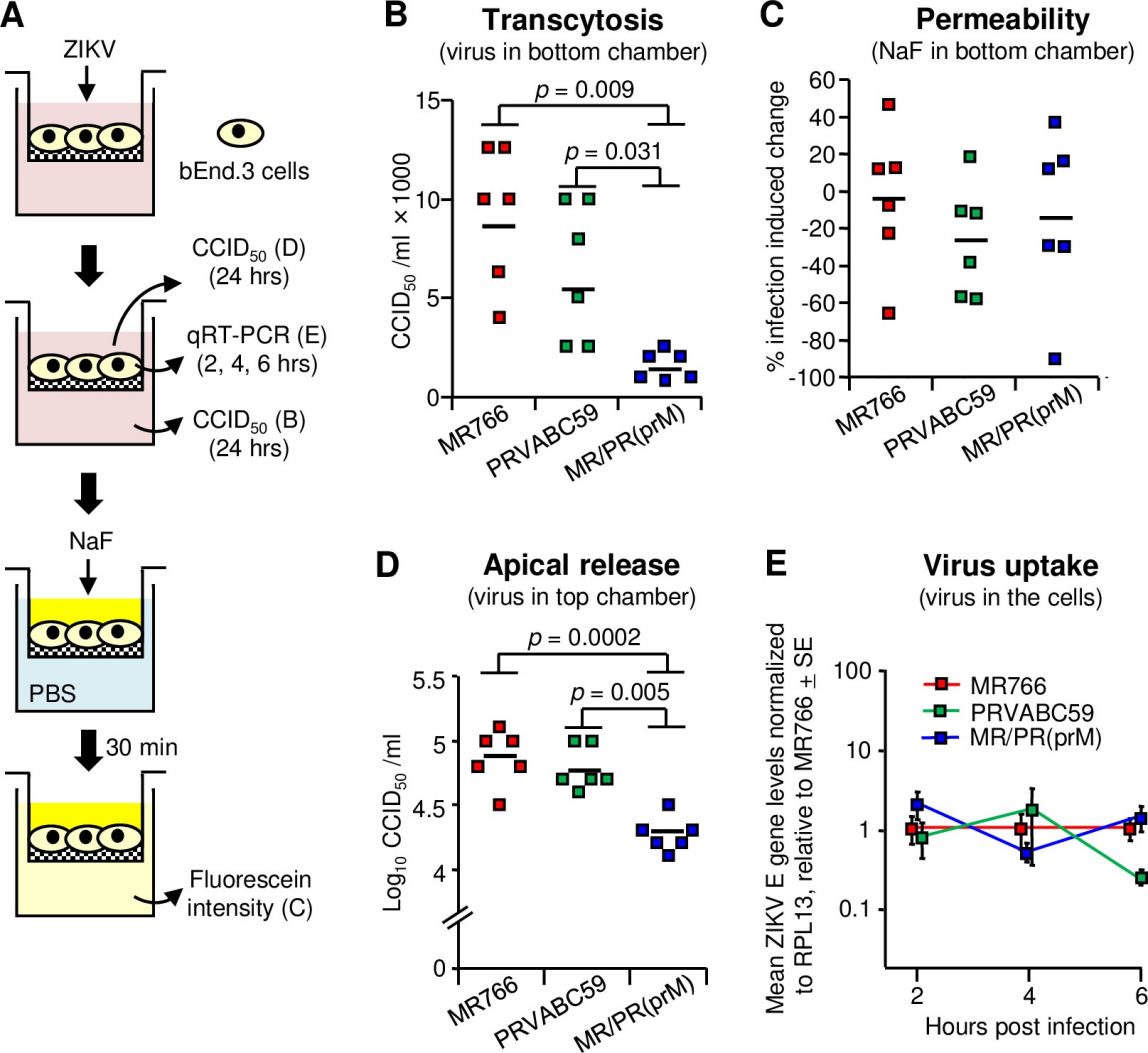
Viral transcytosis and uptake in bEnd.3 cells. (A) *In vitro* Transwell setup. Mouse endothelial cells (bEnd.3) were seeded onto the luminal side of the Transwell insert. After the bEnd.3 monolayers reached confluence, MR766, PRVABC59 or MR/PR(prM) viruses were added to the top well at a MOI of 1. After 2, 4 and 6 hrs, viral RNA levels in the cells were determined by qRT-PCR, and after 24 hrs, virus titers in the upper and lower chambers were determined by CCID_50_ assays. After collecting the culture medium from the upper and lower chambers at 24 hrs, NaF was added to the top wells and PBS to the bottom chambers; after 30 min, samples from the bottom chambers were analyzed by fluorometer. (B) ZIKV levels in the lower chambers. The medium in the lower chamber was collected and virus titers were determined. Kolmogorov-Smirnov tests were used for statistics. Data were obtained from two independent experiments. The horizontal line indicates the mean. (C) The percent of virus-induced permeability. The percent virus-induced permeability was calculated relative to a linear standard curve where “no virus control” was 0% and “no cell control” was 100%. *T*-test was used for statistical analysis. (D) ZIKV levels in the upper chambers. The medium in the upper chambers was collected and virus titers were determined. Kolmogorov-Smirnov tests were used for statistical analyses. (E) Viral uptake by bEnd.3 cells. bEnd.3 cells were inoculated at a MOI of 1 and incubated for 2, 4 and 6 hrs. The cells were washed three times with PBS, trypsinized, washed three times with PBS, and dissolved in TRIzol (n = 4–9 replicates per virus per time point). Uninfected bEnd.3 cells were used as controls (n = 3). ZIKV RNA levels in the cells were determined by qRT-PCR and normalized to RPL13 mRNA levels and graphed relative to MR766 for each time point. Data were obtained from three independent experiments.

A recent study reported that ZIKV actually entered the brain in IFNAR^-/-^ mice via a process of caveola-mediated transcytosis rather than by disruption of the BBB [61]. Transcytosis involves transport of infectious virus particles across an intact brain endothelial cell layer [61,92]. This report is consistent with our IFNAR^-/-^ mouse observations in which high levels of brain virus were seen (Fig 1B and 1C, 2 and 4 dpi) before increased BBB permeability was evident (Fig 1E, 6 dpi). To assess whether MR766, PRVABC59 and MR/PR(prM) might show different ability to transcytose across brain endothelial cells, a Transwell system was set up using the BALB/c mouse-derived, bEnd.3 brain endothelial cell line, which has been frequently used in BBB models (Fig 6A) [89–91]. MR766 was determined to be significantly better at crossing the cell layer than MR/PR(prM) (Fig 6B, *p* = 0.009). PRVABC59 also showed significantly better transcytosis than MR/PR(prM) (*p* = 0.031), with levels not significantly different from MR766 (Fig 6B). No detectable infectious virus could be detected from bEnd.3 cells by growth kinetic assays (S8A Fig), Western blots (S8B Fig) and immune-plaque assays (S8C Fig). In addition, no significant differences in the permeability of the bEnd.3 cell monolayers were found after inoculation with MR766, PRVABC59 and MR/PR(prM) (Fig 6C). The percentage of live bEnd.3 cells at 24 hrs after ZIKV inoculation (the same time point as the transcytosis assay) was also similar for MR766, PRVABC59 and MR/PR(prM) (S8D Fig), suggesting that these viruses did not induce different levels of monolayer damage. The reduced transcytosis into the bottom chamber seen for MR/PR(prM) (Fig 6B) was not due to increased MR/PR(prM) apical release into the top chamber; in fact apical release was significantly reduced (Fig 6D). Levels of internalized virus were also not significantly different for the three viruses at 2, 4 and 6 hrs after virus inoculation (Fig 6E). Swapping the prM of MR766 for the prM of PRVABC59 (to generate MR/PR(prM)) thus significantly reduced transcytosis across the endothelial cell layer when compared with MR766.

### Surface hydrophobicity and positive charge correlate with transcytosis

**Fig 7:**
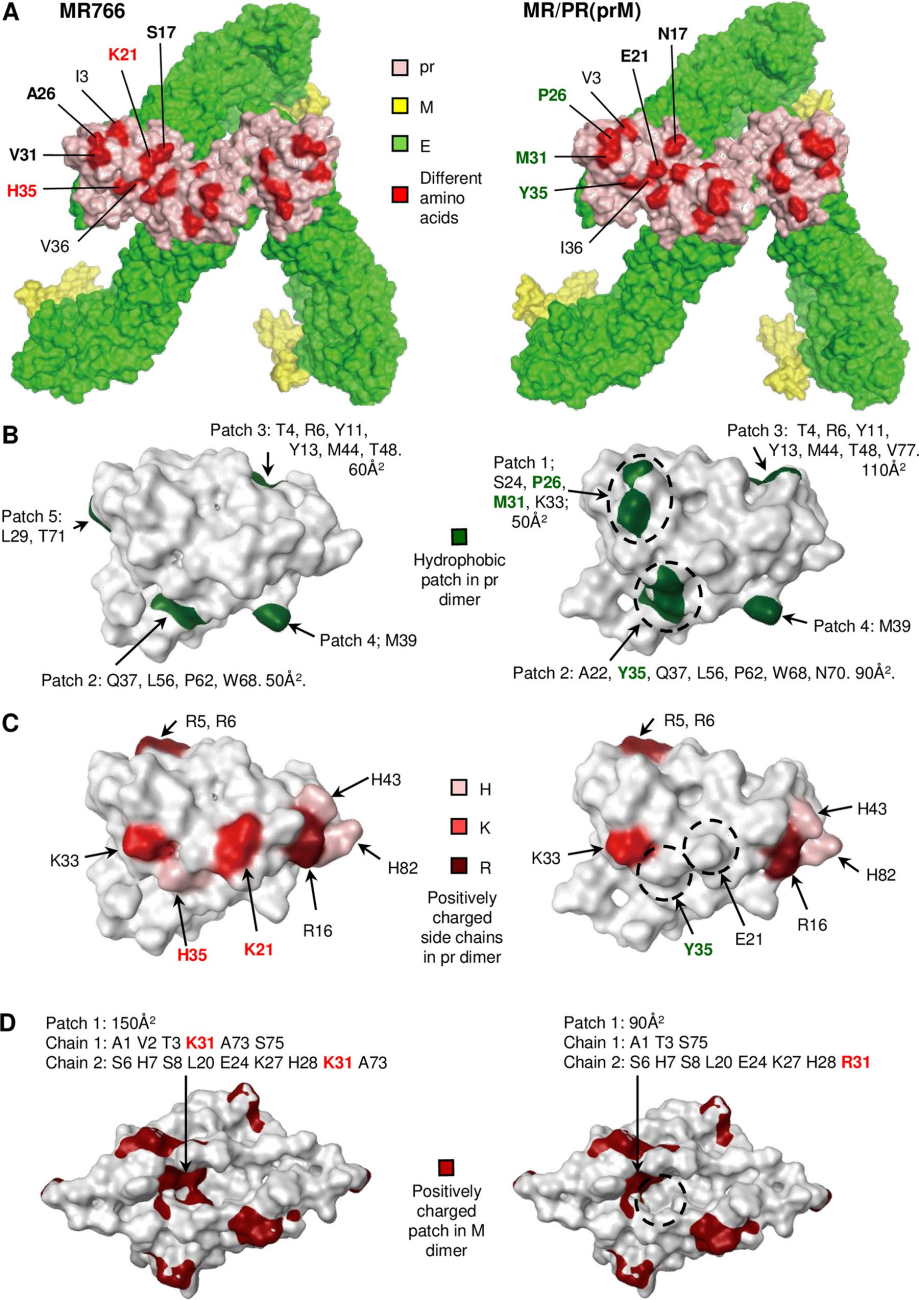
Surface hydrophobicity and positively charge on the prM proteins of MR766 and MR/PR(prM). (A) Molecular model showing the top surface of the prME trimer in the immature virus. The homology models based on the pr structure (PDB accession code: 5U4W) was generated by MOE homology modeler. The pr, M and E proteins are shown in light pink, yellow and green, respectively. Different amino acids in the pr protein between MR766 and MR/PR(prM) are colored red on the trimetric molecular structure. Amino acids (in text) are indicated for one of the pr proteins in the trimer. Bold text indicates amino acids that have side chains exposed on the surface (position 17, 21, 26, 31 and 35; see S2 Table), with font color corresponding to the amino acids highlighted in (B) and (C) (green–hydrophobic patch, red–positively charged). (B) Patch analysis shows that the pr protein of PRVABC59 has an additional hydrophobic patch due to the A26P and V31M substitutions (Patch 1, 50Å^2^) and an enlarged hydrophobic patch (50 to 90Å^2^) due to the H35Y substitution (Patch 2) (dashed circles). The total increase in hydrophobic surface area was 90Å^2^. A further increase is evident for Patch 3 (60 to 110Å^2^), although this patch sits on the side of the trimeric spike. The threshold of the patch area was 50Å^2^. (C) Analyses of positively charged residues on the surface of the pr protein show that the K21E and H35Y substitutions in MR/PR(prM) result in the loss of two positively charged residues exposed (see S2 Table) on the surface of the pr protein (dashed circles). Light pink–histidine (H). Red–Lysine (K). Dark red–Arginine (R). (D) Surface positively charged patches on the M dimer. The patch analysis shows the M protein of MR/PR(prM) (or PRVABC59) decreased a positively charged patch due to the K31R substitution (150 to 90Å^2^) (dashed circles). The M structure of MR766 was homology-modelled based on the structure (PDB accession code: 5IZ7). Top view of M dimer relative to the viral surface is shown. The threshold of the patch area was 50Å^2^. The default MOE settings were used for protein surface patch analysis.

The data so far argues that up to 10 amino acids in the prM protein of MR766 (Fig 3A, MR766 vs. MR/PR(prM)) are important drivers of the high neuroinvasiveness of MR766 via the promotion of BBB transcytosis. Of the 7 amino acids that differ in the pr proteins between MR766 and MR/PR(prM) (Fig 7A, colored in red on the molecular structure of the trimer), 5 amino acids have side-chains with percentage accessible surface areas (ASA(S)) greater than 36% (Fig 7A, bolded residues). The ASA(S) percentage was calculated relative to Gly-X-Gly, where amino acid X was set at 100% surface exposure, given the smallest of possible side chains in the neighboring two glycine residues. An amino acid with an ASA(S) percentage above 36% is deemed to be exposed and accessible for surface interaction(s) [93]. These 5 amino acids increased the number and size of hydrophobic patches and reduced the number of surface exposed positively charged amino acid side chains in MR/PR(prM) when compared with MR766 (Fig 7 and S2 Table). Specifically, the pr protein of MR/PR(prM) had one extra and one enlarged surface hydrophobic patch arising from the A26P, V31M (Patch 1) and H35Y (Patch 2) substitutions, respectively (Fig 6B, dashed ovals). In addition, the H35Y and K21E substitutions resulted in the loss of two surface-accessible positively charged side chains in MR/PR(prM) (Fig 7C, dashed circles).

Of the three amino acids that differ in the M proteins between MR766 and MR/PR(prM), the K31R substitution results in a decrease in the area of a surface exposed positively charged patch in MR/PR(prM), when compared with MR766 (Fig 7D, dashed circle). The other two M protein substitutions, V45A and V47A, are located in the transmembrane domain and are thus not exposed on the virus surface. We propose that the increased surface hydrophobicity and/or the decreased surface positive charge in prM may be responsible for the reduced ability of MR/PR(prM) to transcytosis when compared with MR766 (see Discussion).

The levels of transcytosis for MR766 and PRVABC59 were not significantly different and the level of transcytosis for MR/PR(prM) was significantly lower than for PRVABC59 (Fig 6B). The E protein of MR766 (and thus also MR/PR(prM)) has fewer and smaller surface exposed positive patches and more surface exposed hydrophobic patches when compared with PRVABC59 (S9 Fig). We thus similarly propose that these surface features (but now in E) may be responsible for the reduced ability of MR/PR(prM) to transcytosis when compared with PRVABC59. The reason why PRVABC59 is less virulent than MR766 in mice thus appears not to be due to significantly reduced transcytosis, but rather due to a significantly reduced ability to replicate in adult mouse brain (Figs 4C, 4D and S15C). PRVABC59 is also less neurovirulent than MR766 after i.c. infection of neonates (S10 Fig). These differences in neurovirulence are likely due to other differences between these two viruses [94–96].

To confirm that the E protein of PRVABC59 confers increased neuroinvasiveness, the viral RNA levels in brains were compared after s.c. infection with MR766 or MR/PR(E) (S11 Fig). The viral RNA levels in the brains were higher in MR/PR(E)-infected mice than MR766-infected mice (*p* = 0.0002 by repeated measure ANOVA for 1 to 4 dpi, S11 Fig), suggesting more viruses crossed into the brains. The MR/PR(E) infection caused rapid weight loss (*p* = 0.037 by repeated measure ANOVA for 2 to 5 dpi, Fig 2D) compared to MR766-infected mice. The mean survival time of MR/PR(E)-infected mice (6 days) was shorter than that of MR766 (6.8 days, *p* = 0.008 by log-rank test, Fig 2C). These data thus confirm that the E protein of PRVABC59 (S9 Fig) promotes neuroinvasiveness, consistent with the aforementioned view that increased positive charge increases transcytosis.

## Discussion

The prM protein emerged to be a major determinant of the virulence of MR766 in adult IFNAR^-/-^ mice. The prM substitution in MR/PR(prM) imparted a pronounced reduction in the capacity to enter the brain (neuroinvasiveness) whilst having only a minimal non-significant impact on the ability to replicate in the brain. Neuroinvasiveness in this ZIKV mouse model involves virus crossing the BBB via a process of transcytosis [61], and we illustrate herein that MR/PR(prM) was less able than MR766 to transcytose across a brain endothelial cell barrier *in vitro*. The difference in virulence between MR766 and MR/PR(prM) could thus be mapped to one or more of the 10 amino acid differences in the prM proteins.

Out of the 10 amino acids, four substitutions in pr (residues 21, 26, 31 and 35) and one in the M protein (residue 31) led to decreased positive charge and increased hydrophobicity on the exposed surface of the virion, and this may account for the reduced transcytosis of MR/PR(prM) when compared with MR766. Promotion of transcytosis by positive charges on nanoparticles and liposomes have been independently reported in a variety of systems [97–104] and may reflect positive charge-based interactions with cellular receptors or negatively charged membrane of the BBB [101,102,105]. A K21T substitution in the pr protein of dengue virus significantly reduced the positive charged-based interaction with KDEL receptors, an interaction required for efficient dengue virus egress from the cell [106]. The KDEL receptors are also required for efficient egress of Japanese encephalitis virus [107] and have also been associated with transcytosis in non-viral systems [108,109]. Efficient secretion of dengue virus requires binding of the pr protein to class II ADP-ribosylation factors (Arf4 and Arf5), which are required for KDEL receptor trafficking [110]. The K21E substitution (MR766 to MR/PR(prM)) similarly resulted in the loss of an accessible positively charged residue (Fig 7C), although the role of the KDEL receptors and Arf4/5 in ZIKV infections is currently unknown. Alternatively, increased positive charge on the virus may foster adsorptive interactions with the negatively charged luminal surface of the BBB to facilitate transcytosis [101,103,105,111]. Isoelectric point (pI) calculations suggested the pI of the prME virus particles of MR766, were higher than that of MR/PR(prM) (S12A Fig). To provide experimental evidence for an increased positive charge on MR766, the migration patterns of MR766, PRVABC59 and MR/PR(prM) virus particles in gel electrophoresis was compared as described previously [112]. At pH 6.0 and pH 6.4, MR766 migrated less toward the anode than MR/PR(prM), indicating that MR766 was less negatively charged (i.e. more positively charged) than MR/PR(prM) (S12B Fig, aroows). These data are consistent with the pI calculations (S12A Fig) and the aforementioned charge-based mechanism for the increased transcytosis of MR766.

Two substitutions in the pr protein of pathogenic WNV NY99 strain (WNV_NY99_), V22I and S72L, were reported to be associated with decreased virulence, with no significant human outbreaks ever occurring with the WNV Kunjin strain (WNV_KUNJIN_), which contains I22 and L72 [17]. These substitutions in the pr protein also increase the area of accessible hydrophobic patches on the surface of WNV_KUNJIN_ when compared with WNV_NY99_ (see S13 Fig). The mechanism whereby such increases in accessible surface hydrophobic patches might decrease virulence remains unclear, with prM cleavage not implicated either herein (Fig 3) or for WNV [17]. Conceivably, increased hydrophobicity may promote virus aggregation [113,114] or trafficking to lysosomes [115,116] and/or may increase association/retention [117,118] in the cholesterol-rich hydrophobic membranes of caveolae during transcytosis [119,120].

The reverse chimera, PR/MR(prM), in which the prM of PRVABC59 was replaced with the prM of MR766, was constructed to see whether the prM of MR766 could confer increased virulence to PRVABC59. After s.c. infection with 1 × 10^4^ of PR/MR(prM), 100% of the IFNAR^-/-^ mice survived (S14 Fig), indicating that virulence was not significantly increased. The similar result was seen in the previous report, in which anti-IFNAR-antibody pretreated C57BL/6J mice survived after s.c. infection with PR/MR(prM/E) yet MR766-infected mice died [121]. This observation is perhaps consistent with previous work in which CprM chimeras between MR766 and an Asian strain (BR15) showed that MR/BR(CprM) resulted in decreased cell death *in vitro* over MR766, whereas the reverse chimera, BR/MR(CprM), did not change cell death when compared with BR15 [15]. Although prM may be a virulence factor for MR766 and other African isolates, other genes are likely to be responsible for the lower virulence of PRVABC59 and perhaps also other Asian strains of ZIKV [87,122–124].

A potential limitation of the current study is that we have a limited understanding of cell tropism and replication competencies in different cells *in vivo* in IFNAR^-/-^ mice and whether these are affected by the different strains and chimeras. To try and address this issue, primary IFNAR^-/-^ MEFs [125], IFNAR^-/-^ embryo-derived neurons and bone-marrow-derived macrophages were infected with the parental viruses and MR/PR(prM). Although replication of these viruses was very similar in type I IFN deficient Vero cells (Fig 2B), in these primary cells MR/PR(prM) generally replicated better than PRVABC59, but less well than MR766 (S15A Fig). The viral titers in the primary neurons infected with MR766 *in vitro* correlated with brain titers after i.c. infection with MR766 *in vivo* (*p* = 0.025, S15B Fig). However, we were unable to find any other correlation between replication in these primary cells *in vitro* and brain viral titers or viremias *in vivo* (S15B, S15D and S15E Fig). Some level of evidence exists that these cell types are infected with ZIKV [126–129]. However, although the primary cells used herein can be cultured *in vitro*, virus replication in these cells did not reflect virus replication *in vivo*, suggesting such experiments may not be overly informative. In support of the latter contention, we have previously shown that differences in replication of two ZIKV strains in IFNAR^-/-^ MEFs did not reflect their replication in various tissues in IFNAR^-/-^ mice [125]. *In vivo* virulence similarly did not correlate with *in vitro* growth in a related study using chimeric WNVs [130].

Conventional wisdom would argue that the virulence of MR766 in IFNAR^-/-^ mice is the result of the extensive passage of this virus in brains of suckling wild-type mice [23,28]. However, comparing the sequences of multiple ZIKVs does not provide any support for the notion that the aforementioned 10 amino acid substitutions in the prM proteins arose from passaging in suckling mice. These 10 amino acids are conserved in nearly all African genotype ZIKVs, whether passaged in suckling mouse brain or not (Table 1). Furthermore, both African ZIKVs, ArD 41525 (DAKAR41525) and ArD 41524 (DAKAR41524) without a passage history in suckling mice, were 100% lethal in adult IFNAR^-/-^ mice after intraperitoneal and s.c. infection [86,131] and showed 100% homology with MR766 for the aforementioned 10 amino acids (Table 1). The African genotype virus ArD 41519 (DAKAR41519) was not passaged in suckling mice, but was nevertheless more virulent than PRVABC59 in adult CD-1/ICR mice after i.c. infection [20] and had a similarly conserved prM protein. Furthermore, MP1751 (Uganda, 1962) was passaged up to 3 times in suckling mouse brains, was 100% conserved in the 10 amino acids, and was 100% lethal in adult IFNAR^-/-^ mice after s.c. infection, whereas PRVABC59 was again not lethal [67,132]. The virulence characteristic of MR766 is thus shared with other African genotype viruses and would therefore appear to be independent of the passage history in suckling mice. One might thus infer that the higher virulence of African genotype viruses is a legitimate characteristic of these viruses [133]. The date presented herein argues that up to 10 of the aforementioned conserved amino acids in prM contribute to this virulence by increasing the capacity to cross the BBB, although our *in vivo* data is limited to IFNAR^-/-^ mice or IFNAR^+/-^ fetuses.

**Table 1:** The 10 amino acid differences between prM proteins of African and Asian ZIKV isolates. Amino acid coloring corresponds with that used in Fig 7. SM, suckling mouse; ?, Unknown; NGS, next-generation sequencing.

Genotype	Strain	Accession number	Source	Country	Year	Passage history	pr								M		
							3	17	21	26	31	35	36	31	45	47
**AFRICAN**	**MR766**	LC002520	Monkey	Uganda	1947	146×SM, 1 × C6/36, 1 × VeroE6	I	S	K	A	V	H	V	K	V	V
	MP1751	KY288905	Mosquito	Uganda	1962	3×SM,? × VeroE6	I	S	K	A	V	H	V	K	V	V
	IbH_30656	HQ234500	Human	Nigeria	1968	21×SM, 1 × Vero	I	S	K	V	V	H	V	K	V	V
	ArB 1362	KF383115	Mosquito	Central African Republic	1968	Not available	I	S	K	A	V	H	V	K	A	V
	ArD7117	KF383116	Mosquito	Senegal	1968	Not available	I	S	K	A	V	H	V	K	V	V
	ARB13565	KF268948	Mosquito	Central African Republic	1976	Not available	I	S	K	A	V	H	V	K	A	V
	ARB7701	KF268950	Mosquito	Central African Republic	1976	Not available	I	S	K	A	V	H	V	K	A	V
	ARB15076	KF268949	Mosquito	Central African Republic	1980	Not available	I	S	K	A	V	H	V	K	A	V
	ArD_41519	HQ234501	Mosquito	Senegal	1984	1 × AP61, 2 × C6/36	I	S	K	A	V	H	V	K	V	V
	ArD_41525	KU955591	Mosquito	Senegal	1984	1 × AP61, 1 × C6/36, 3 × VeoE6	I	S	K	A	V	H	V	K	V	V
	ArD_41524	KX601166	Mosquito	Senegal	1984	1 × AP61, 1 × C6/36, 2 × VeoE6	I	S	K	A	V	H	V	K	V	V
	ArD128000	KF383117	Mosquito	Senegal	1997	Not available	I	S	K	A	V	H	V	K	A	V
	ArD157995	KF383118	Mosquito	Senegal	2001	Not available	I	S	K	A	V	H	V	M	V	V
**ASIAN**	**PRVABC59**	KU501215	Human	Puerto Rico	2015	No passage. NGS of patient sample	V	N	E	P	M	Y	I	R	A	A
	P6-740	HQ234499	Mosquito	Malaysia	1966	6×SM, 1 × Vero, 1 × BHK, 1 × C6/36	V	S	E	P	V	Y	I	R	A	A
	Yap/Micronesia	EU545988	Human	Micronesia	2007	No passage. Sequence only	V	S	E	P	M	Y	I	R	A	A
	FSS13025	JN860885	Human	Cambodia	2010	1 × Vero	V	S	E	P	M	Y	I	R	A	A
	H/PF/2013	KJ776791	Human	French Polynesia	2013	3 × Vero	V	N	E	P	M	Y	I	R	A	A
	BeH819966	KU365779	Human	Brazil	2015	? × C6/36	V	N	E	P	M	Y	I	R	A	A
	FLR	KU820897	Human	Colombia	2015	1 × C6/36	V	N	E	P	M	Y	I	R	A	A
	Brazil-ZKV2015	KU497555	Human	Brazil	2015	No passage. NGS of amniotic fluid.	V	N	E	P	M	Y	I	R	A	A
	Natal RGN	KU527068	Human	Brazil	2015	Not available	V	N	E	P	M	Y	I	R	A	A
	BeH818305	KU729218	Human	Brazil	2015	? × C6/36	V	N	E	P	M	Y	I	R	A	A
	Rio-U1	KU926309	Human	Brazil	2016	1 × VeroE6	V	N	E	P	M	Y	I	R	A	A
	Rio-S1	KU926310	Human	Brazil	2016	1 × VeroE6	V	N	E	P	M	Y	I	R	A	A
	ZIKVNL00013	KU937936	Human	Suriname	2016	Vero	V	N	E	P	M	Y	I	R	A	A
	ZKA-16-291	KX827309	Human	Singapore	2016	Not available	V	S	E	P	M	Y	I	R	A	A
	ZIKV/Hu/NIID123/2016	LC219720	Human	Vietnam	2016	1 × C6/36, 4 × Vero	V	S	E	P	M	H	I	R	A	A

Numerous studies have attempted to explain how and why CZS is largely restricted to ZIKV of Asian origin [16,29,57,86,125,133–141]. Our data suggest that MR766 and PRVABC59 have comparable neuroinvassive properties (Figs 5A, 5D and 6B), but that MR766 is more neurovirulent than PRVABC59 (Figs 4C, 4D and S10). If African genotype viruses are generally more neurovirulent [20,25], one might speculate that they would more reliably induce miscarriage and/or fetal demise [123,142–144], thereby reducing the likelihood of births of viable infants with CZS (S16 Fig) [29,140,141,145,146].

## Supporting information

S1 Fig MR766 viruses with mutations/deletions in the NDT N-linked glycosylation site in the E protein. (A) Amino acid sequence alignment. The sequences of the N-linked glycosylation motif in the E protein (positions 154–156, amino acids NDT) are shown in yellow. The amino acid (**N**) that is glycosylated is shown in bold. Survival data were derived from the indicated references and from (D), with mutant MR766-NIID viruses that were constructed using rZIKV-MR766/pMW119-CMVP and primers (see S1 Table) as previously described [34,147]. (1)–Annamalai AS, Pattnaik A, Sahoo BR, Muthukrishnan E, Natarajan SK, Steffen D, et al. Zika Virus Encoding Nonglycosylated Envelope Protein Is Attenuated and Defective in Neuroinvasion. J Virol. 2017;91(23):e01348-17. (2)–Carbaugh DL, Baric RS, Lazear HM. Envelope Protein Glycosylation Mediates Zika Virus Pathogenesis. J Virol. 2019;93(12):e00113-19. (B) Growth kinetics of MR766 and recombinant viruses in Vero cells. None of the viruses had defects in *in vitro* replication. The cells were infected at a MOI of 0.01, and the supernatant was collected at the indicated times. The viral titres were determined by CCID_50_ assays on Vero cells. Each data point represents the average of 4 wells. (C) Endoglycosidase analysis on E protein. Each virus was sucrose purified from the supernatant of infected Vero cells and treated with PNGase F under non-denaturing conditions (for 24 hrs at 37°C). The PNGase F-treated and non-treated viruses were separated by SDS-PAGE and probed with 4G2 monoclonal antibody (Absolute Antibody Ltd., Oxford, UK). Glycosylation of NDT E protein (digested by PNGase F) and non-glycosylation of MR766-NIID E protein (not digested by PNGase F) were confirmed. (D) Survival rate of IFNAR^-/-^ mice after s.c. infection with indicated viruses. Mice were infected with 1 × 10^4^ PFU of MR766-NIID-Δ156–161 (n = 6), MR766-NIID-Δ153–156 (n = 6) or MR766-NIID-NDT (n = 6) and monitored until 14 dpi. Comparison of Kaplan-Meier survival curves between groups was performed by log-rank analysis. Comparisons for MR766-NIID-NDT versus either MR766-NIID-Δ153–156 or MR766-NIID-Δ156–161, *p* = 0.0009. (E) Mean percent weight change relative to day 0 after s.c. infection with 1 × 10^4^ PFU of MR766-NIID-Δ156–161 (n = 6), MR766-NIID-Δ153–156 (n = 6) or MR766-NIID-NDT (n = 6). Statistical analysis was performed by repeat measure ANOVA for 1 to 6 dpi. (F) Possible association of glycan loop deletion in MR766 virulence. Although infections with all glycosylated viruses were associated with mortality, for four viruses (red), increased survival was associated with deletions of several amino acids in the glycan loop. Mutations in NDT to NDI (MR766-NIID) or ADT (m2MR) were associated with no change in virulence or a change to 100% survival, respectively, compared to the original MR766 virus. (G) The glycan loop sits near the fusion loop (residue 99-RGWGNGCGLFG-109, magenta). Deletion/mutations in the glycan loop (residues 146-SQHSGMIVNDTGHETDE-162, red) may affect virulence independently of glycosylation [36].
(TIF)

S2 Fig Age of infected IFNAR^-/-^ mice and virus titers and cytokine levels in serum after MR766 or PRVABC59 subcutaneous infection of IFNAR^-/-^ mice. (A) Age of individual IFNAR^-/-^ mice infected s.c. with MR766 or PRVABC59. IFNAR^-/-^ mice were infected with 1 × 10^6^ PFU of PRVABC59 (n = 6), 1 × 10^4^ PFU of MR766 (n = 10) or PRVABC59 (n = 12), 1 × 10^3^ PFU of MR766 (n = 6) or PRVABC59 (n = 9), or 1 × 10^2^ PFU of MR766 (n = 6) or PRVABC59 (n = 6). Kolmogorov-Smirnov tests were used for statistical analyses. (B) No correlation between mouse age and survival time after infection was seen. Significance was determined by Spearman’s correlation test. (C) No correlation between mouse age and peak viremia titers was seen. Significance was determined by Spearman’s correlation test. (D) Viremias of individual IFNAR^-/-^ mice infected s.c. with MR766 or PRVABC59. IFNAR^-/-^ mice were infected with 1 × 10^6^ PFU of PRVABC59 (n = 6), 1 × 10^4^ PFU of MR766 (n = 10) or PRVABC59 (n = 6), 1 × 10^3^ PFU of MR766 (n = 10) or PRVABC59 (n = 16), or 1 × 10^2^ PFU of MR766 (n = 6) or PRVABC59 (n = 6). Serum viral titers were determined by CCID_50_ assays. Percentages in the upper right corner of each graph indicate the percent survival (see Fig 1A). Numbers at the bottom of each graph indicate the mean viremia titers ± SE for 1–6 dpi. Limit of detection was 2 log_10_CCID_50_/ml indicated by the horizontal dashed line. Statistical analyses to compare viremia titers were performed using repeated-measures ANOVAs. (E) Serum cytokine levels in MR766- and PRVABC59-infected IFNAR^-/-^ mice. IFNAR^-/-^ mice were infected s.c. with 1 × 10^4^ PFU of MR766 or PRVABC59, and serum samples were collected at 1, 3 and 5 dpi (n = 3). TNFα, IL-6 and IL-1β levels in the serum samples were analyzed using a mouse cytokine magnetic 20-plex panel kit (Thermo Fisher Scientific, Tokyo, Japan) and a Luminex 100/200 system (Luminex Corporation, Tokyo, Japan). The concentration of each cytokine was determined by comparison to a standard curve according to the manufacturer’s instructions. Six uninfected mice were used as a control. There were no statistically significant differences in serum cytokine levels between MR766- and PRVABC59-infected mice by 2-way ANOVA.
(TIF)

S3 Fig Survival and viremia for IFNAR^-/-^ mice infected with chimeric MR766/PRVABC59 viruses. (A) Survival of IFNAR^-/-^ mice infected s.c. with 1 × 10^4^ PFU of MR766, PRVABC59 or MR/PR(prM) (n = 6). Comparisons of Kaplan-Meier survival curves between the different groups were performed by log-rank analyses. Comparisons for MR766 versus either PRVABC59 or MR/PR(prM), *p* = 0.0009. (B) Viremia of individual mice infected with wild-type or chimeric viruses. Eight to fourteen-week-old IFNAR^-/-^ mice were infected s.c. with 1 × 10^4^ PFU of MR766 (n = 16), PRVABC59 (n = 18) or each chimeric virus (n = 6–13). Serum samples were prepared to quantify viral titer by CCID_50_ assays. Numbers in the upper right corner of each graph indicate % survival, and those at the bottom of each graph indicate the mean viremia titer ± SE for 1–6 dpi. Limit of detection was 2 log_10_CCID_50_/ml indicated by the horizontal dashed line. (C) No correlation between % survival and mean viremia titer for 1–6 dpi. Significance was determined by Pearson’s correlation test. (D) No correlation between % survival and mean viremia titer at 6 dpi. Significance was determined by Pearson’s correlation test. (E) No correlation between % survival and peak viremia titer. Significance was determined by Pearson’s correlation test.
(TIF)

S4 Fig Virulence of MR/PR(pr) and MR/PR(M) in IFNAR^-/-^ mice. (A) Schematic representation of viral genes of MR/PR(pr) and MR/PR(M). (B) Growth kinetics of wild-type and chimeric viruses. Vero cells were infected at a MOI of 0.01, and the supernatants were collected at the indicated times. The viral titres were determined by plaque assay on Vero cells [34]. Each data point represents the average of 4–8 wells. (C) Survival of IFNAR^-/-^ mice infected s.c. with 1 × 10^4^ PFU of MR766 (n = 3), PRVABC59 (n = 3) or each chimeric virus (n = 6). The indicated comparison was between MR766 and MR/PR(pr) (*p* = 0.039; log-rank test). (D) Mean percent weight change relative to day 0 after s.c. infection with 1 × 10^4^ PFU of MR766 (n = 3), PRVABC59 (n = 3) or each chimeric virus (n = 6). (E) Viremia of individual mice infected with wild-type or chimeric viruses. Numbers in the upper right corner of each graph indicate % survival, and those at the bottom of each graph indicate the mean viremia titer ± SE for 1–6 dpi. Limit of detection was 2 log_10_CCID_50_/ml indicated by the horizontal dashed line.
(TIF)

S5 Fig Amino acid substitutions in the E protein. (A) Amino acid sequences of N-linked glycosylation motif (positions 154–156, amino acids NDT) and the residue at position 330 in the E protein. The virus stocks of MR766, MR/PR(C), MR/PR(prM), MR/PR(NS4B/NS5), MR/PR(NS5), MR/PR(pr) and MR/PR(M) do not have N-linked glycosylation motif due to the T156I substitution. The amino acid (**N**) at 154 that is glycosylated is shown in bold. V/L shows the mixture of V and L. (B) Survival and N-linked glycosylation motif of viruses recovered from infected mouse sera at 4 dpi. The substitutions are shown in yellow compared to each virus stock. I/T shows the mixture of I and T. The amino acid (**N**) at 154 that is glycosylated is shown in bold. (C) Survival and the amino acid position 330 in the E protein. The amino acid of PRVABC59 was the mixture of V and L (V/L) as previously reported [33] (see Materials and Methods). L at position 330 was reported to be associated with reduced virulence of PRVABC59 in AG129 mice compared to V [33]. To see whether amino acid at position 330 correlated with survival, the viral sequences of PRVABC59 and MR/PR(E), in which the E of MR766 was replaced with the E of PRVABC59, recovered from infected mouse sera at 4 dpi was determined (n = 2). In all mice infected with PRVABC59, the amino acid was a mixture of V and L, and all mice survived (Fig 2C). In all mice infected with MR/PR(E), the amino acid was L, yet all mice reached the ethical endpoint for euthanasia by 6 dpi (Fig 2C). Thus, in this context, L at position 330 did not correlate with survival.
(TIF)

S6 Fig Fetal weights. (A) Fetal weights. Pregnant IFNAR^-/-^ mice mated with C57BL/6J male mice were infected s.c. with 1 × 10^4^ PFU of MR766, PRVABC59 or MR/PR(prM) at E15.5 (n = 3 dams in each group). The infected dams were sacrificed and the fetuses were weighted at E18.5. + indicates the weights of fetuses with detectable virus in the brain (see Fig 5A);—indicates the weight of fetuses with no detectable virus in the brain. Five fetuses from uninfected dam were used as control (Cont.). Kolmogorov-Smirnov test or *t*-test were used for statistical analyses. (B) No correlation between fetal weights and ZIKV RNA levels in fetal brains was seen. Significance was determined by Spearman’s correlation test. **Conclusion.** The reduced neuroinvasiveness in fetuses of MR/PR(prM)-infected dams was not due to the greater fetal growth with no difference in the fetal weights among MR766, PRVABC59 and MR/PR(prM).
(TIF)

S7 Fig *In vitro* BBB permeability assay. (A) The experimental procedure of the *in vitro* BBB system. bEnd.3 cells (murine brain microvascular endothelial cell line, 5 × 10^4^ /Transwell) and C8D1A cells (murine astrocyte cell line, 1 × 10^4^ /Transwell) were seeded onto the top and the bottom side of the Transwell filter, respectively. After the bEnd.3 monolayers had grown to confluence, MR766, PRVABC59 or MR/PR(prM) were added to the top well at a MOI of 1. After 48 hrs, NaF was added to the top wells and PBS to the bottom wells; after 30 min, samples from the bottom wells were analyzed by fluorometer. The % virus-induced permeability was calculated relative to a linear standard curve where “no virus control” was 0% and “no cell control” was 100%. (B) The mean % virus-induced permeability. Results are from five independent experiments. *T*-test was used for statistical analysis. **Infection of C8D1A cells.** (C) The experimental procedure for (D)–(I). C8D1A cells were seeded in 12-well plates at a density of 2 × 10^5^ /well and infected at a MOI of 1 for MR766, PRVABC59 or MR/PR(prM). The supernatant was collected at 48 hrs after infection (n = 4 replicates). Virus titers were determined by CCID_50_ assays. Uninfected cells were used as a control (n = 4). *T*-tests were used for statistical analyses. (D) Viral titers in the supernatant of C8D1A cells. (E) qRT-PCR of ZIKV RNA using prM primers 48 hrs after infection of C8D1A cells. The infected C8D1A cells were dissolved in TRIzol (n = 4). Uninfected C8D1A cells were used as controls (n = 4). ZIKV RNA levels in the cells were determined by qRT-PCR and normalized to RPL13 mRNA levels. Statistical analyses were performed by *t*-tests or Kolmogorov-Smirnov tests. (F) As in (E) using ZIKV E primers. (G) As in (E) for TNFα mRNA. (H) As in (E) for IL-1β mRNA. (I) As in (E) for IL-6 mRNA. (J) Growth kinetics of wild-type and chimeric viruses in C8D1A cells. C8D1A cells were infected at a MOI of 0.1, and the supernatants were collected at the indicated times. The viral titres were determined by CCID_50_ assays. Each data point represents the average of 6 wells. Data were obtained from two independent experiments. Growth in this astrocyte cell line did not correlate with virulence in mice [15]. **Conclusion.** The standard Transwell BBB (A, B) was thus compromised by the significantly higher replication (D–F, J) and subsequently higher cytokine levels (G, I) of MR766 in the C8D1A cells when compared with PRVABC59.
(TIF)

S8 Fig No detectable replication of ZIKVs in bEnd.3 cells. (A) Growth kinetics of MR766, PRVABC59 and MR/PR(prM) in bEnd.3 cells. bEnd.3 cells were inoculated at a MOI of 1. The supernatants were collected at the indicated times, and viral titres in the supernatant were determined by CCID_50_ assays. Each data point represents the average of 4 wells. (B) Western blot of cells inoculated with MR766, PRVABC59 and MR/PR(prM). bEnd.3 cells or VeroE6 cells were inoculated with MR766, PRVABC59 or MR/PR(prM) at a MOI of 1 or of 0.01, respectively, and incubated for 24 or 72 hrs. The cells were lysed in RIPA buffer (0.1% SDS, 1% NP40, 0.1% sodium deoxycholate, 140 mM NaCl, 1 mM EDTA and Protease Inhibitor Cocktail, Roche, Mannheim, Germany). The lysate was analyzed using hyper-immune mouse sera against ZIKV and horseradish peroxidase-conjugated anti-mouse IgG antibody (in Materials and Methods). M, marker. 1, uninfected. 2, MR766. 3, PRVABC59. 4, MR/PR(prM). 5, MR/PR(C). 6, MR/PR(E). 7, purified MR766 (in Materials and Methods). (C) Immuno-plaque assay of MR766, PRVABC59 and MR/PR(prM). bEnd.3 cells or VeroE6 cells were inoculated with MR766, PRVABC59 or MR/PR(prM) at a MOI of 2.5 or of 1, respectively, and incubated for 72 hr. The immuno-plaque assay was performed as described previously [148]. Each image is representative of duplicate wells. (D) The percent of live bEnd.3 cells after ZIKV inoculation. The cells were inoculated with MR766, PRVABC59 or MR/PR(prM) at a MOI of 1 and incubated for 24 hr, and the numbers of live or dead cells were counted. Each data point represents the average of 8 wells. Data were obtained from two independent experiments.
(TIF)

S9 Fig Surface patches on the E dimer of MR766 and PRVABC59. (A) The structure of dimeric E-M complex in the mature virus. The homology models based on 5IZ7 were generated by MOE homology modeler. The M and E proteins are shown in yellow and green, respectively. The 17 different amino acids in the E protein between MR766 and PRVABC59 are shown as red spheres. The positions of each different residue are indicated on one of the E proteins in the dimer. (B) The positively charged patch analysis shows the E protein of PRVABC59 has additional positively charged patches and an increase in the areas of other positively charged patches. These are due both to amino acid substitutions and slightly different folding and side chain exposures; see D below. The top view of the E dimers relative to the viral surface is shown. The threshold for a patch area was 50 Å^2^. The default MOE settings were used for protein surface patch analyses. (C) The hydrophobic patch analysis shows the E protein of PRVABC59 has fewer exposed hydrophobic patches. The top view of the E dimers relative to the viral surface is shown. The threshold and MOE settings as for B. (D) The amino acids contributing to each patch; substitutions highlighted in red.
(TIF)

S10 Fig Survival of neonatal mice after intracranial infection with MR766 or PRVABC59. Neonatal C57BL/6J mice were infected i.c. with 1 × 10^4^ PFU of MR766 (n = 5) or PRVABC59 (n = 3) and monitored for 7 dpi. Comparison of Kaplan-Meier survival curves between groups was performed by log-rank analysis.
(TIF)

S11 Fig ZIKV RNA levels in the brain after s.c. infection with MR766 or MR/PR(E) in IFNAR^-/-^ mice. Ten to twelve-week-old IFNAR^-/-^ mice were s.c. infected with 1 × 10^4^ PFU of MR766 or MR/PR(E) (n = 4–5 mice per group). At indicated time points, mice were sacrificed and perfused with 50 ml of cold PBS through the left ventricle of the heart to flush out intravascular viruses and the brains were collected. ZIKV RNA levels in brains were determined by qRT-PCR with the prM gene-specific primers and normalized to RPL13 mRNA levels. Three uninfected mouse brains were used to determine the limit of detection (10^−6.29^, indicated by the horizontal dashed line). Statistical analyses were performed using repeated-measures ANOVAs.
(TIF)

S12 Fig pI of each virus. (A) The calculated pI value. The pI based on the sequences or protein structures was calculated using ExPASy ProtParam tool (http://www.expasy.ch/tools/protparam.html), Prot pi Protein Tool (https://www.protpi.ch/Calculator/ProteinTool) or MOE (Ver. 2019.01). In MOE, the sequence-based pI was calculated from the protein sequence according to [149] (see also http://isoelectric.org/www_old/files/practise-isoelectric-point.html) and structure-based pI was calculated using a modification to the algorithm of [149] in that individual amino acid group pKa’s estimated from 3D coordinates and local hydrogen bond networks are used. The pKa values are calculated according to the PROPKA algorithm [150]. The default MOE settings were used for the pI calculation. All three tools suggested that pI of the prME of MR766 was higher than the prME of MR/PR(prM). (B) Gel electrophoresis of purified MR766, PRVABC59 and MR/PR(prM) viruses in pH 6.0 or pH 6.4 buffers. The electrophoresis was performed as previously described [112], with modifications. Briefly, the surface of the 8 × 4 cm GelBond File (Lonza, Tokyo, Japan) was coated with 7–8 ml of 0.8% agarose (Takara Bio Inc. Shiga, Japan). The gels were electrophoresed in citric acid phosphate buffer (pH 6.0 or pH 6.4) [54] at 90 volts (ATTA model AE-8750) for 60 min at 4°C. After the electrophoresis, proteins were stained with Coomassie brilliant blue. Arrows indicate the position of MR766 or MR/PR(prM).
(TIF)

S13 Fig Hydrophobic surface patches of West Nile virus pr protein. The homology model of WNV_NY99_ and WNV_KUNJIN_ pr proteins based on the structure (PDB accession code: 3C6E) was generated with MOE homology modeler. Protein surface patch analysis shows that the pr protein of the WNV_KUNJIN_ has larger hydrophobic patches (Patch 1 and Patch 2) (dashed ovals) exposed on the top of the trimeric spike. The increase in hydrophobic patch area was 60Å^2^ or 26% (80 + 150 versus 110 + 180). Patches 3 and 6 face other pr proteins, and Patch 5 faces the E protein in the trimeric spike. The 2 different amino acid residues (positions 22 and 72) in the pr protein between WNV_NY99_ and WNV_KUNJIN_ are colored in green. The threshold of the patch area was 50Å^2^. The default MOE settings were used for protein surface patch analysis. (A) Top view or (B) side view of pr protein relative to the virion surface.
(TIF)

S14 Fig The virulence of PR/MR(prM) in IFNAR^-/-^ mice. (A) Schematic representation illustrating prM gene swapping to generate PR/MR(prM). Recombinant molecular clones of PR/MR(prM) were constructed using the modified infectious-subgenomic-amplicons method and primers (see S1 Table) as previously described [151]. (B) Growth kinetics of PR/MR(prM) in Vero cells. Vero cells were infected at a MOI of 0.01. The viral titers were determined by CCID_50_ assays on Vero cells. Each data point represents the mean titer of 4 wells. (C) Survival of adult IFNAR^-/-^ mice infected with 1 × 10^4^ PFU of PR/MR(prM) by i.c. (n = 11) or s.c. (n = 11) infection. The i.c. or s.c. infection with 1 × 10^4^ PFU of PR/MR(prM) resulted in 27.3% or 100% survival in IFNAR^-/-^ mice, respectively. These survival data were not significantly different from PRVABC59 (data from Figs 2C and 4D): 41.7% (*p* = 0.62) or 100% (*p* = 1.00) survival after i.c. or s.c. infection, respectively. However, these survival data were significantly different from MR766 (data from Figs 2C and 4D): 0% (*p*<0.0001) for both i.c. and s.c. infections (by log-rank analysis). (D) Mean percent weight change relative to day 0 after infection with 1 × 10^4^ PFU of PR/MR(prM) by i.c. (n = 11) or s.c. (n = 11). (E) Tissue titers after i.c. infection with 1 × 10^4^ PFU of PR/MR(prM) (n = 4). Organs were harvested at 4 dpi, and viral titers were determined by CCID_50_ assays. The brain viral titer for PR/MR(prM)-infected mice was 2.4 logs lower than that for MR766 (*p* = 0.0004 by *t*-test, Fig 4C). Limit of detection was 0.83 log_10_CCID_50_/g indicated by the horizontal dashed line. (F) As in E after s.c. infection with 1 × 10^4^ PFU of PR/MR(prM) (n = 5). Organs were harvested at 6 dpi. (G) Viremia of individual IFNAR^-/-^ mice infected s.c. with 1 × 10^4^ PFU of PR/MR(prM) (n = 11). These viremias levels for 1–7 dpi were not significantly different from those obtained after s.c. infection with 1 × 10^4^ PFU of MR766 or PRVABC59 (data from S2B Fig) (*p* = 0.69 or *p* = 0.34, respectively; repeated-measures ANOVA). Limit of detection was 2 log_10_CCID_50_/ml indicated by the horizontal dashed line. **Conclusion.** PR/MR(prM) retained the low virulence of PRVABC59 and did not adopt the high virulence of MR766. The prM genes of MR766 therefore did not confer virulence to PRVABC59.
(TIF)

S15 Fig Growth kinetics of wild-type and chimeric viruses in primary cells from IFNAR^-/-^ mice. (A) Primary neurons, macrophages and MEFs were infected at a MOI of 0.1 or 1 and the supernatants were collected at the indicated times. The viral titers were determined by CCID_50_ assays on Vero cells. Each data point represents the mean titer of 3–7 wells. Statistical analyses were performed using repeated-measures ANOVAs: * *p* < 0.05; ** *p* < 0.01. (B) The correlation diagrams between mean viral titer in neurons and mean brain titer after i.c. infection. Ten to fourteen-week-old IFNAR^-/-^ mice were i.c. infected with 1 × 10^4^ PFU of MR766, PRVABC59 or MR/PR(prM) and the brains were harvested at 1, 2 or 3 dpi (n = 4–5 mice per group). The viral titers were determined by CCID_50_ assays. The brain titers at 4 dpi are the same as those shown in Fig 4C. Significance was determined by Pearson’s correlation test. (C) Viral titers in brains after i.c. infection. (D) No significant correlation between mean viral titer in macrophages and mean viremia after s.c infection. Significance was determined by Pearson’s correlation test. (E) No significant correlation between mean viral titer in MEFs and mean viremia after s.c. infection. Significance was determined by Pearson’s correlation test.
(TIF)

S16 Fig Summary of main findings and conclusions. (TIF)

S1 Table Primers used in the study. References: (1) Lanciotti RS, Kosoy OL, Laven JJ, Velez JO, Lambert AJ, Johnson AJ, et al. Genetic and serologic properties of Zika virus associated with an epidemic, Yap State, Micronesia, 2007. Emerg Infect Dis. 2008;14:1232–39; (2) Dang JW, Tiwari SK, Qin Y, Rana TM. Genome-wide Integrative Analysis of Zika-Virus-Infected Neuronal Stem Cells Reveals Roles for MicroRNAs in Cell Cycle and Stemness. Cell Rep. 2019;27(12):3618–28 e5; (3) Gardner J, Anraku I, Le TT, Larcher T, Major L, Roques P, et al. Chikungunya virus arthritis in adult wild-type mice. J Virol. 2010;84:8021–32; (4) Castro-Jorge LA, Pretto CD, Smith AB, Foreman O, Carnahan KE, Spindler KR. A Protective Role for Interleukin-1 Signaling during Mouse Adenovirus Type 1-Induced Encephalitis. J Virol. 2017;91:e02106-16; (5) Wang P, Dai J, Bai F, Kong KF, Wong SJ, Montgomery RR, et al. Matrix metalloproteinase 9 facilitates West Nile virus entry into the brain. J Virol. 2008;82:8978–85.
(DOCX)

S2 Table The solvent-accessible surface area of amino acid side-chains (ASA(S)) of the seven amino acids that differ between the pr proteins of MR766 and MR/PR(prM). The ASA(S) of each amino acid was calculated with the ASA Calculator program (MOLSIS Inc., Tokyo, Japan) operated by MOE, using the homology model of the trimer structure of prME (PDB accession code: 5U4W). Solvent accessibility classes were defined relative to values calculated for all residue types in a Gly-X-Gly tripeptide (both backbone and side-chain conformations of X being fully extended). Three solvent accessibility classes are defined: buried (< 9%), partially exposed (< 36%) and exposed (> 36%) [93]. Color coding as in Fig 7 (red–positively charged, green–hydrophobic patch).
(DOCX)
