# Supplementary figures and images for: Neuroinvasiveness of the MR766 strain of Zika virus in IFNAR-/- mice maps to prM residues conserved amongst African genotype viruses
Source: PLoS Pathog. 2021 Jul 26;17(7):e1009788. doi: 10.1371/journal.ppat.1009788 (PMC8341709; doi:10.1371/journal.ppat.1009788)

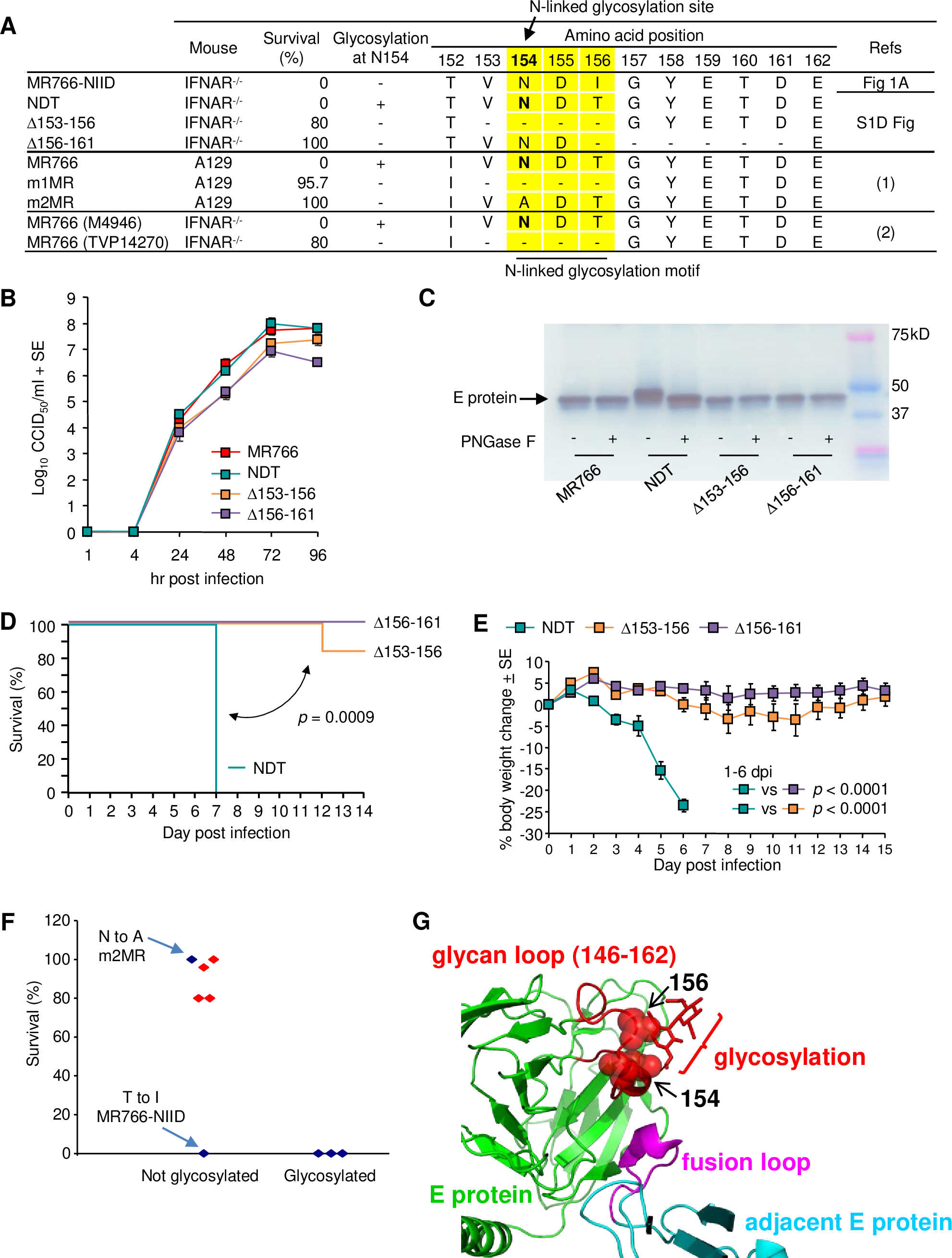

Supplement: S1 Fig — (A) Amino acid sequence alignment. The sequences of the N-linked glycosylation motif in the E protein (positions 154–156, amino acids NDT) are shown in yellow. The amino acid (N) that is glycosylated is shown in bold. Survival data were derived from the indicated references and from (D), with mutant MR766-NIID viruses that were constructed using rZIKV-MR766/pMW119-CMVP and primers (see S1 Table) as previously described [34,147]. (1)–Annamalai AS, Pattnaik A, Sahoo BR, Muthukrishnan E, Natarajan SK, Steffen D, et al. Zika Virus Encoding Nonglycosylated Envelope Protein Is Attenuated and Defective in Neuroinvasion. J Virol. 2017;91(23):e01348-17. (2)–Carbaugh DL, Baric RS, Lazear HM. Envelope Protein Glycosylation Mediates Zika Virus Pathogenesis. J Virol. 2019;93(12):e00113-19. (B) Growth kinetics of MR766 and recombinant viruses in Vero cells. None of the viruses had defects in in vitro replication. The cells were infected at a MOI of 0.01, and the supernatant was collected at the indicated times. The viral titres were determined by CCID50 assays on Vero cells. Each data point represents the average of 4 wells. (C) Endoglycosidase analysis on E protein. Each virus was sucrose purified from the supernatant of infected Vero cells and treated with PNGase F under non-denaturing conditions (for 24 hrs at 37°C). The PNGase F-treated and non-treated viruses were separated by SDS-PAGE and probed with 4G2 monoclonal antibody (Absolute Antibody Ltd., Oxford, UK). Glycosylation of NDT E protein (digested by PNGase F) and non-glycosylation of MR766-NIID E protein (not digested by PNGase F) were confirmed. (D) Survival rate of IFNAR-/- mice after s.c. infection with indicated viruses. Mice were infected with 1 × 10^4^ PFU of MR766-NIID-Δ156–161 (n = 6), MR766-NIID-Δ153–156 (n = 6) or MR766-NIID-NDT (n = 6) and monitored until 14 dpi. Comparison of Kaplan-Meier survival curves between groups was performed by log-rank analysis. Comparisons for MR766-NIID-NDT versus either MR766-NIID [file ppat.1009788.s001.tif]

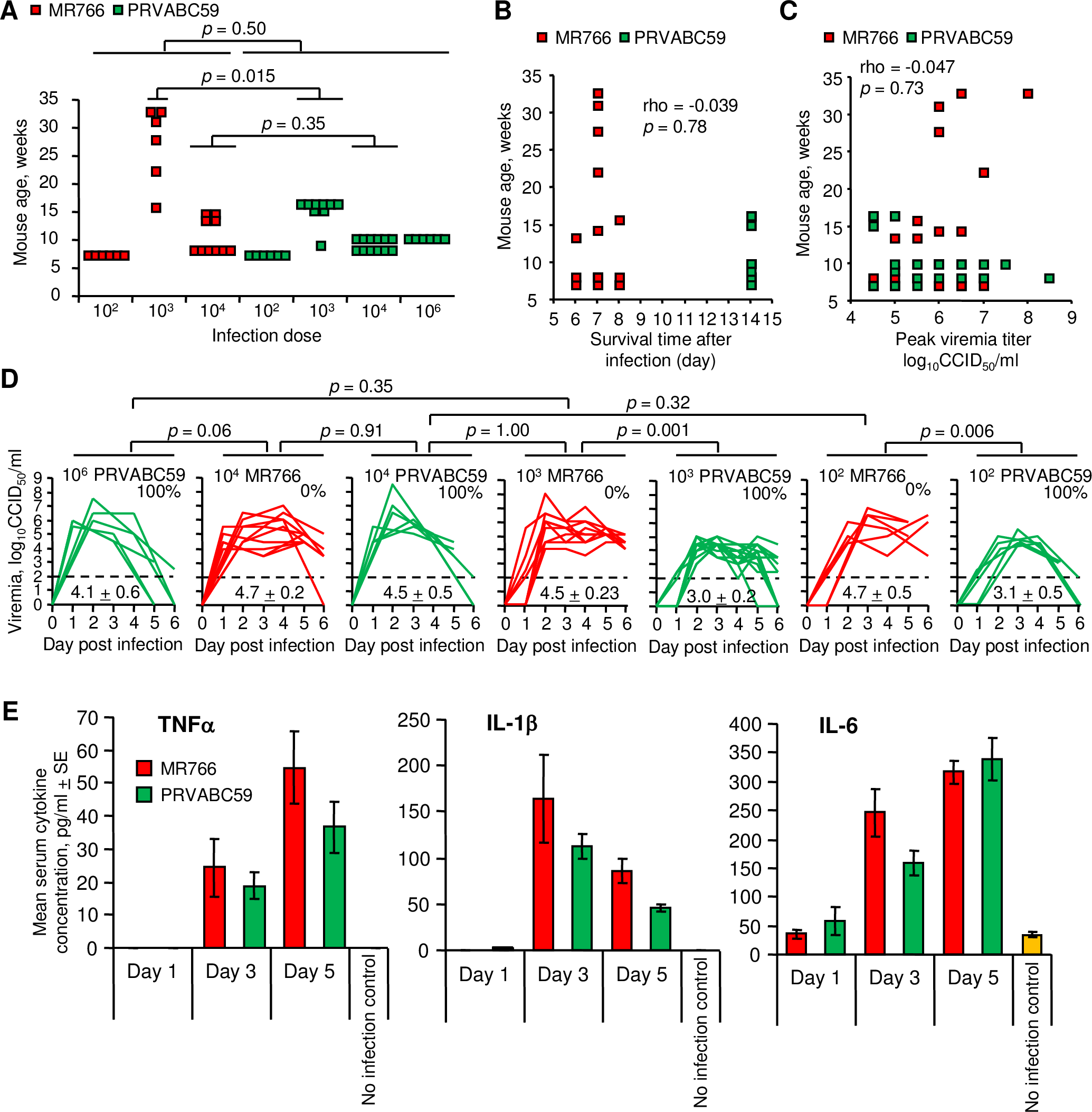

Supplement: S2 Fig — (A) Age of individual IFNAR-/- mice infected s.c. with MR766 or PRVABC59. IFNAR-/- mice were infected with 1 × 10^6^ PFU of PRVABC59 (n = 6), 1 × 10^4^ PFU of MR766 (n = 10) or PRVABC59 (n = 12), 1 × 10^3^ PFU of MR766 (n = 6) or PRVABC59 (n = 9), or 1 × 10^2^ PFU of MR766 (n = 6) or PRVABC59 (n = 6). Kolmogorov-Smirnov tests were used for statistical analyses. (B) No correlation between mouse age and survival time after infection was seen. Significance was determined by Spearman’s correlation test. (C) No correlation between mouse age and peak viremia titers was seen. Significance was determined by Spearman’s correlation test. (D) Viremias of individual IFNAR-/- mice infected s.c. with MR766 or PRVABC59. IFNAR-/- mice were infected with 1 × 10^6^ PFU of PRVABC59 (n = 6), 1 × 10^4^ PFU of MR766 (n = 10) or PRVABC59 (n = 6), 1 × 10^3^ PFU of MR766 (n = 10) or PRVABC59 (n = 16), or 1 × 10^2^ PFU of MR766 (n = 6) or PRVABC59 (n = 6). Serum viral titers were determined by CCID50 assays. Percentages in the upper right corner of each graph indicate the percent survival (see Fig 1A). Numbers at the bottom of each graph indicate the mean viremia titers ± SE for 1–6 dpi. Limit of detection was 2 log10CCID50/ml indicated by the horizontal dashed line. Statistical analyses to compare viremia titers were performed using repeated-measures ANOVAs. (E) Serum cytokine levels in MR766- and PRVABC59-infected IFNAR-/- mice. IFNAR-/- mice were infected s.c. with 1 × 10^4^ PFU of MR766 or PRVABC59, and serum samples were collected at 1, 3 and 5 dpi (n = 3). TNFα, IL-6 and IL-1β levels in the serum samples were analyzed using a mouse cytokine magnetic 20-plex panel kit (Thermo Fisher Scientific, Tokyo, Japan) and a Luminex 100/200 system (Luminex Corporation, Tokyo, Japan). The concentration of each cytokine was determined by comparison to a standard curve according to the manufacturer’s instructions. Six uninfected mice were used as a control. There were no statistically significant differences in serum [file ppat.1009788.s002.tif]

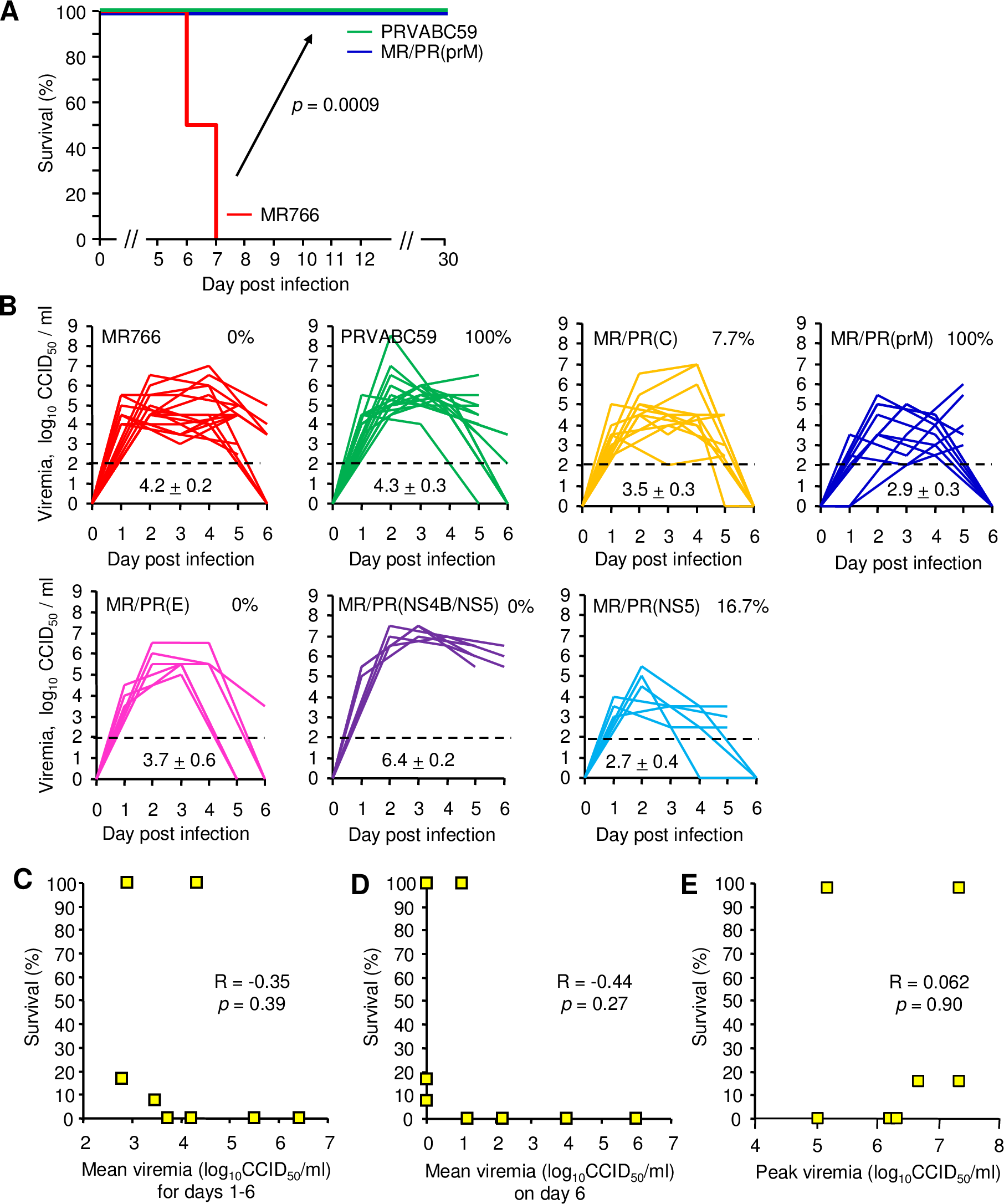

Supplement: S3 Fig — (A) Survival of IFNAR-/- mice infected s.c. with 1 × 10^4^ PFU of MR766, PRVABC59 or MR/PR(prM) (n = 6). Comparisons of Kaplan-Meier survival curves between the different groups were performed by log-rank analyses. Comparisons for MR766 versus either PRVABC59 or MR/PR(prM), p = 0.0009. (B) Viremia of individual mice infected with wild-type or chimeric viruses. Eight to fourteen-week-old IFNAR-/- mice were infected s.c. with 1 × 10^4^ PFU of MR766 (n = 16), PRVABC59 (n = 18) or each chimeric virus (n = 6–13). Serum samples were prepared to quantify viral titer by CCID50 assays. Numbers in the upper right corner of each graph indicate % survival, and those at the bottom of each graph indicate the mean viremia titer ± SE for 1–6 dpi. Limit of detection was 2 log10CCID50/ml indicated by the horizontal dashed line. (C) No correlation between % survival and mean viremia titer for 1–6 dpi. Significance was determined by Pearson’s correlation test. (D) No correlation between % survival and mean viremia titer at 6 dpi. Significance was determined by Pearson’s correlation test. (E) No correlation between % survival and peak viremia titer. Significance was determined by Pearson’s correlation test. (TIF) [file ppat.1009788.s003.tif]

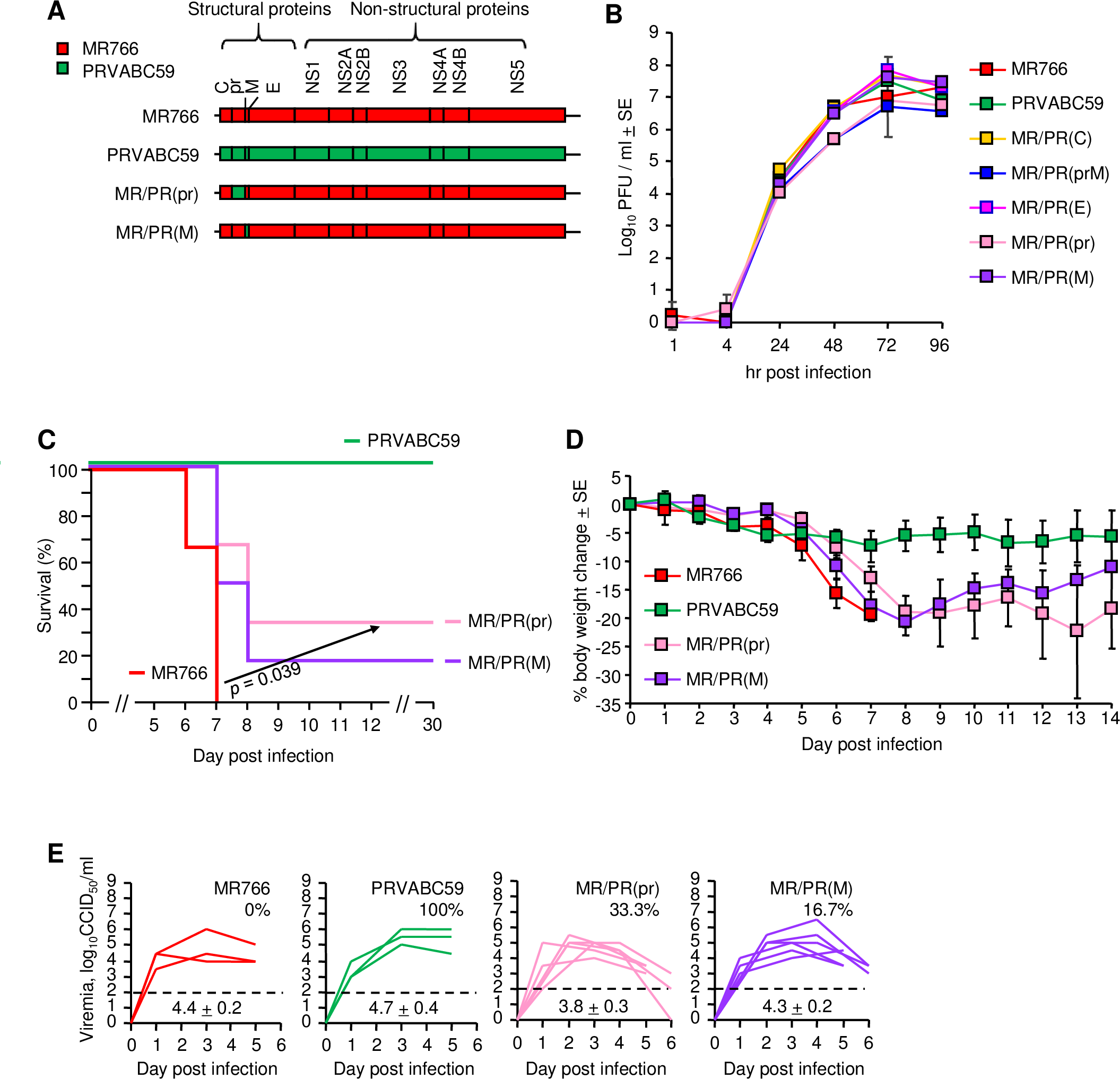

Supplement: S4 Fig — (A) Schematic representation of viral genes of MR/PR(pr) and MR/PR(M). (B) Growth kinetics of wild-type and chimeric viruses. Vero cells were infected at a MOI of 0.01, and the supernatants were collected at the indicated times. The viral titres were determined by plaque assay on Vero cells [34]. Each data point represents the average of 4–8 wells. (C) Survival of IFNAR-/- mice infected s.c. with 1 × 10^4^ PFU of MR766 (n = 3), PRVABC59 (n = 3) or each chimeric virus (n = 6). The indicated comparison was between MR766 and MR/PR(pr) (p = 0.039; log-rank test). (D) Mean percent weight change relative to day 0 after s.c. infection with 1 × 10^4^ PFU of MR766 (n = 3), PRVABC59 (n = 3) or each chimeric virus (n = 6). (E) Viremia of individual mice infected with wild-type or chimeric viruses. Numbers in the upper right corner of each graph indicate % survival, and those at the bottom of each graph indicate the mean viremia titer ± SE for 1–6 dpi. Limit of detection was 2 log10CCID50/ml indicated by the horizontal dashed line. (TIF) [file ppat.1009788.s004.tif]

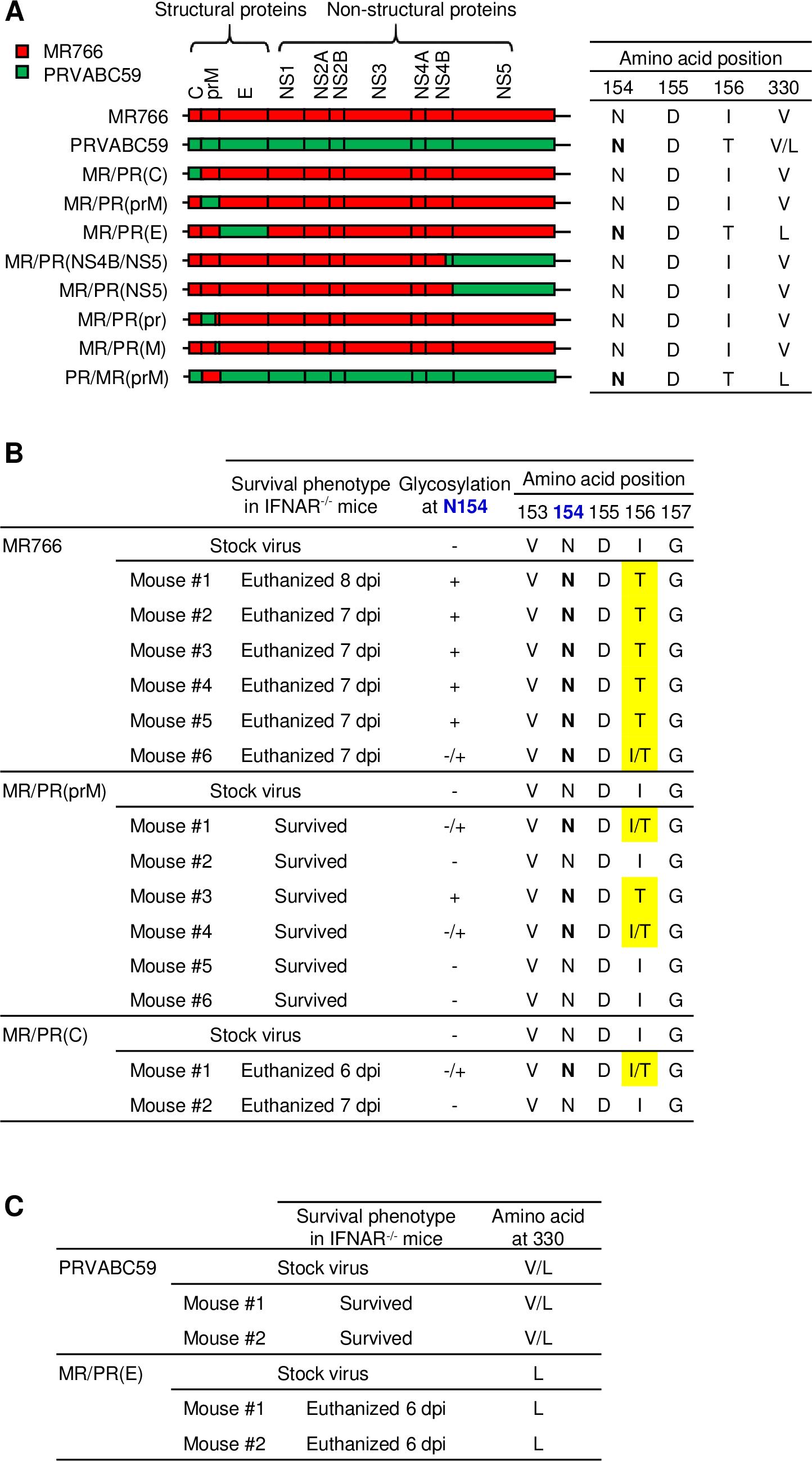

Supplement: S5 Fig — (A) Amino acid sequences of N-linked glycosylation motif (positions 154–156, amino acids NDT) and the residue at position 330 in the E protein. The virus stocks of MR766, MR/PR(C), MR/PR(prM), MR/PR(NS4B/NS5), MR/PR(NS5), MR/PR(pr) and MR/PR(M) do not have N-linked glycosylation motif due to the T156I substitution. The amino acid (N) at 154 that is glycosylated is shown in bold. V/L shows the mixture of V and L. (B) Survival and N-linked glycosylation motif of viruses recovered from infected mouse sera at 4 dpi. The substitutions are shown in yellow compared to each virus stock. I/T shows the mixture of I and T. The amino acid (N) at 154 that is glycosylated is shown in bold. (C) Survival and the amino acid position 330 in the E protein. The amino acid of PRVABC59 was the mixture of V and L (V/L) as previously reported [33] (see Materials and Methods). L at position 330 was reported to be associated with reduced virulence of PRVABC59 in AG129 mice compared to V [33]. To see whether amino acid at position 330 correlated with survival, the viral sequences of PRVABC59 and MR/PR(E), in which the E of MR766 was replaced with the E of PRVABC59, recovered from infected mouse sera at 4 dpi was determined (n = 2). In all mice infected with PRVABC59, the amino acid was a mixture of V and L, and all mice survived (Fig 2C). In all mice infected with MR/PR(E), the amino acid was L, yet all mice reached the ethical endpoint for euthanasia by 6 dpi (Fig 2C). Thus, in this context, L at position 330 did not correlate with survival. (TIF) [file ppat.1009788.s005.tif]

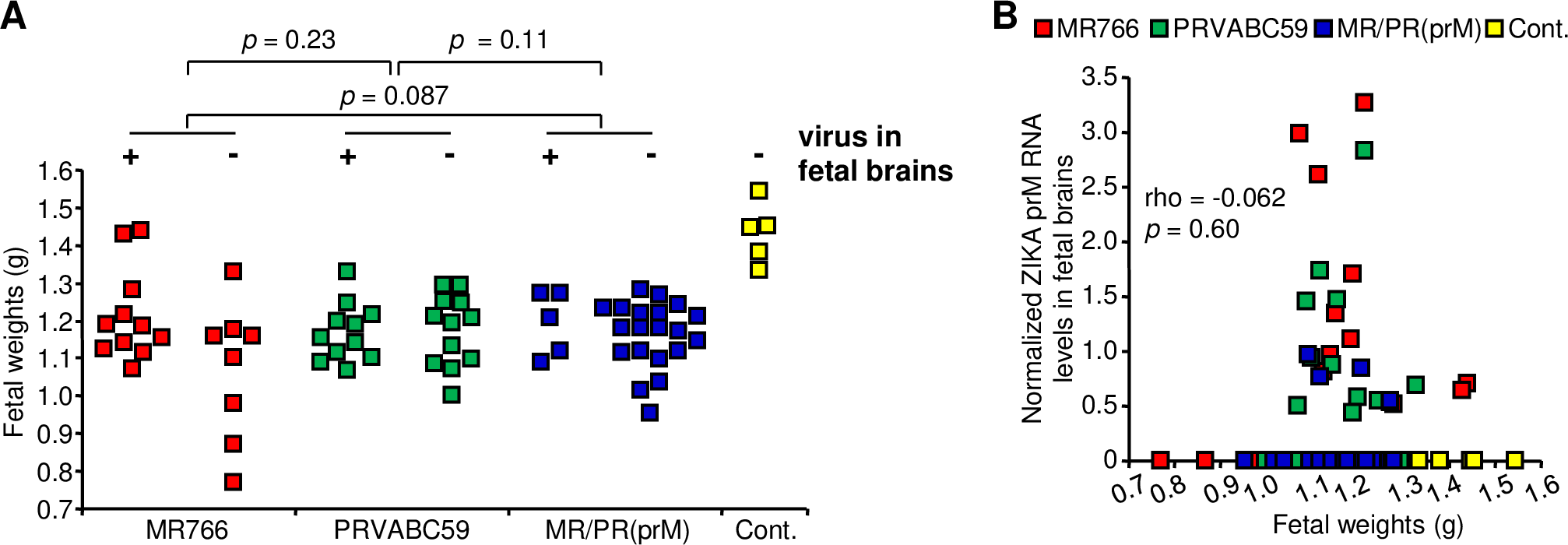

Supplement: S6 Fig — (A) Fetal weights. Pregnant IFNAR-/- mice mated with C57BL/6J male mice were infected s.c. with 1 × 10^4^ PFU of MR766, PRVABC59 or MR/PR(prM) at E15.5 (n = 3 dams in each group). The infected dams were sacrificed and the fetuses were weighted at E18.5. + indicates the weights of fetuses with detectable virus in the brain (see Fig 5A);—indicates the weight of fetuses with no detectable virus in the brain. Five fetuses from uninfected dam were used as control (Cont.). Kolmogorov-Smirnov test or t-test were used for statistical analyses. (B) No correlation between fetal weights and ZIKV RNA levels in fetal brains was seen. Significance was determined by Spearman’s correlation test. Conclusion. The reduced neuroinvasiveness in fetuses of MR/PR(prM)-infected dams was not due to the greater fetal growth with no difference in the fetal weights among MR766, PRVABC59 and MR/PR(prM). (TIF) [file ppat.1009788.s006.tif]

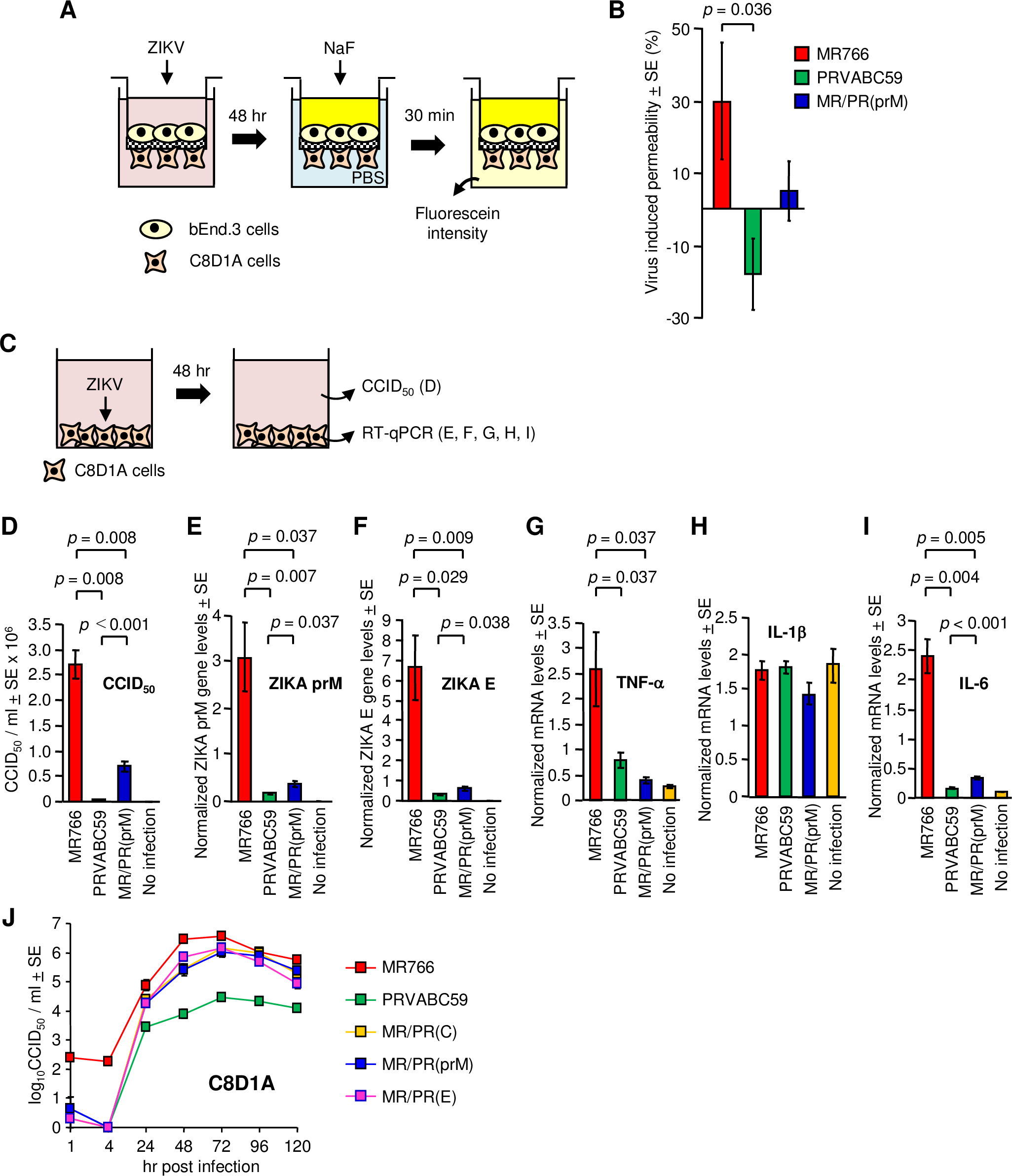

Supplement: S7 Fig — (A) The experimental procedure of the in vitro BBB system. bEnd.3 cells (murine brain microvascular endothelial cell line, 5 × 10^4^ /Transwell) and C8D1A cells (murine astrocyte cell line, 1 × 10^4^ /Transwell) were seeded onto the top and the bottom side of the Transwell filter, respectively. After the bEnd.3 monolayers had grown to confluence, MR766, PRVABC59 or MR/PR(prM) were added to the top well at a MOI of 1. After 48 hrs, NaF was added to the top wells and PBS to the bottom wells; after 30 min, samples from the bottom wells were analyzed by fluorometer. The % virus-induced permeability was calculated relative to a linear standard curve where “no virus control” was 0% and “no cell control” was 100%. (B) The mean % virus-induced permeability. Results are from five independent experiments. T-test was used for statistical analysis. Infection of C8D1A cells. (C) The experimental procedure for (D)–(I). C8D1A cells were seeded in 12-well plates at a density of 2 × 10^5^ /well and infected at a MOI of 1 for MR766, PRVABC59 or MR/PR(prM). The supernatant was collected at 48 hrs after infection (n = 4 replicates). Virus titers were determined by CCID50 assays. Uninfected cells were used as a control (n = 4). T-tests were used for statistical analyses. (D) Viral titers in the supernatant of C8D1A cells. (E) qRT-PCR of ZIKV RNA using prM primers 48 hrs after infection of C8D1A cells. The infected C8D1A cells were dissolved in TRIzol (n = 4). Uninfected C8D1A cells were used as controls (n = 4). ZIKV RNA levels in the cells were determined by qRT-PCR and normalized to RPL13 mRNA levels. Statistical analyses were performed by t-tests or Kolmogorov-Smirnov tests. (F) As in (E) using ZIKV E primers. (G) As in (E) for TNFα mRNA. (H) As in (E) for IL-1β mRNA. (I) As in (E) for IL-6 mRNA. (J) Growth kinetics of wild-type and chimeric viruses in C8D1A cells. C8D1A cells were infected at a MOI of 0.1, and the supernatants were collected at the indicated times. The viral titres were d [file ppat.1009788.s007.tif]

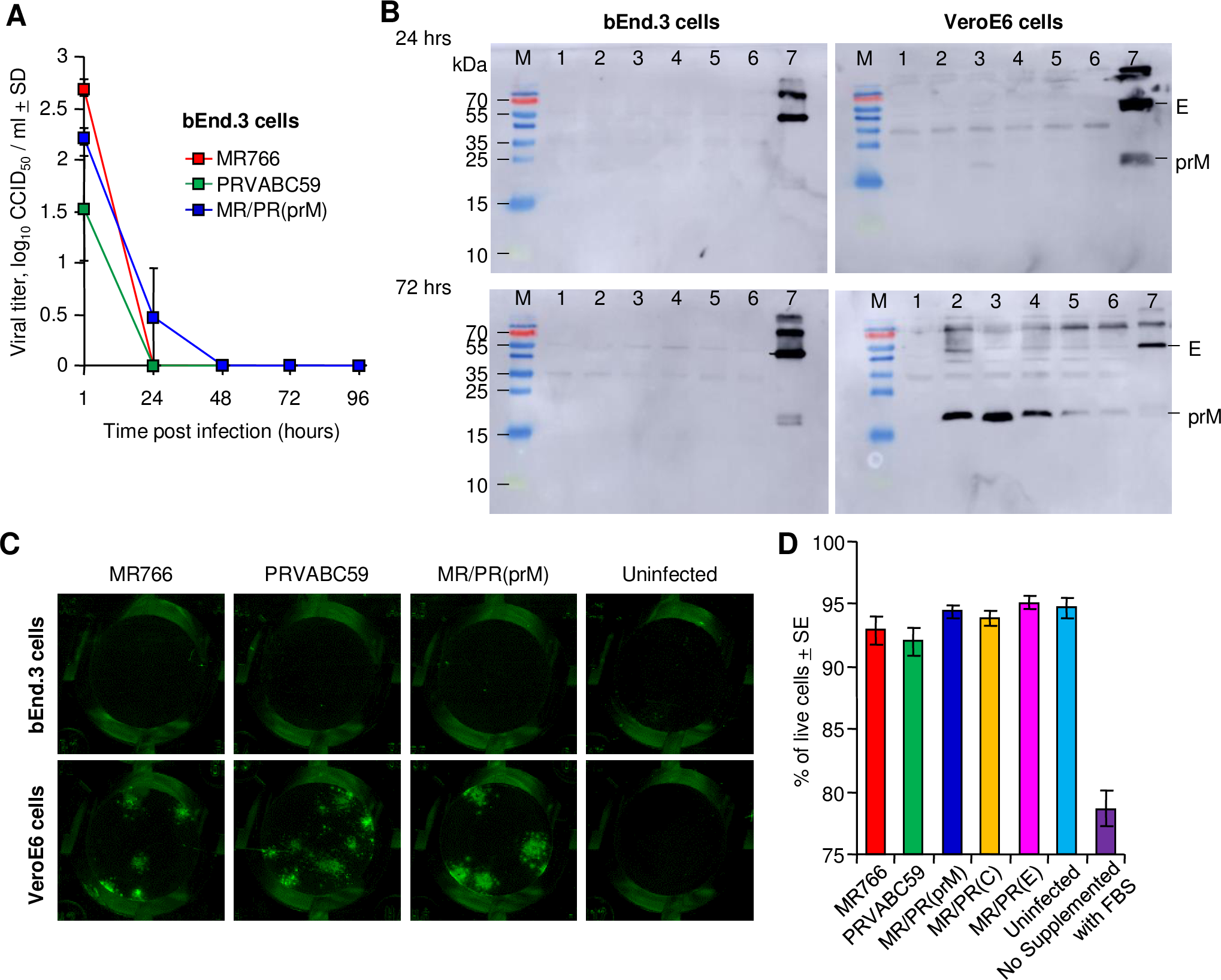

Supplement: S8 Fig — (A) Growth kinetics of MR766, PRVABC59 and MR/PR(prM) in bEnd.3 cells. bEnd.3 cells were inoculated at a MOI of 1. The supernatants were collected at the indicated times, and viral titres in the supernatant were determined by CCID50 assays. Each data point represents the average of 4 wells. (B) Western blot of cells inoculated with MR766, PRVABC59 and MR/PR(prM). bEnd.3 cells or VeroE6 cells were inoculated with MR766, PRVABC59 or MR/PR(prM) at a MOI of 1 or of 0.01, respectively, and incubated for 24 or 72 hrs. The cells were lysed in RIPA buffer (0.1% SDS, 1% NP40, 0.1% sodium deoxycholate, 140 mM NaCl, 1 mM EDTA and Protease Inhibitor Cocktail, Roche, Mannheim, Germany). The lysate was analyzed using hyper-immune mouse sera against ZIKV and horseradish peroxidase-conjugated anti-mouse IgG antibody (in Materials and Methods). M, marker. 1, uninfected. 2, MR766. 3, PRVABC59. 4, MR/PR(prM). 5, MR/PR(C). 6, MR/PR(E). 7, purified MR766 (in Materials and Methods). (C) Immuno-plaque assay of MR766, PRVABC59 and MR/PR(prM). bEnd.3 cells or VeroE6 cells were inoculated with MR766, PRVABC59 or MR/PR(prM) at a MOI of 2.5 or of 1, respectively, and incubated for 72 hr. The immuno-plaque assay was performed as described previously [148]. Each image is representative of duplicate wells. (D) The percent of live bEnd.3 cells after ZIKV inoculation. The cells were inoculated with MR766, PRVABC59 or MR/PR(prM) at a MOI of 1 and incubated for 24 hr, and the numbers of live or dead cells were counted. Each data point represents the average of 8 wells. Data were obtained from two independent experiments. (TIF) [file ppat.1009788.s008.tif]

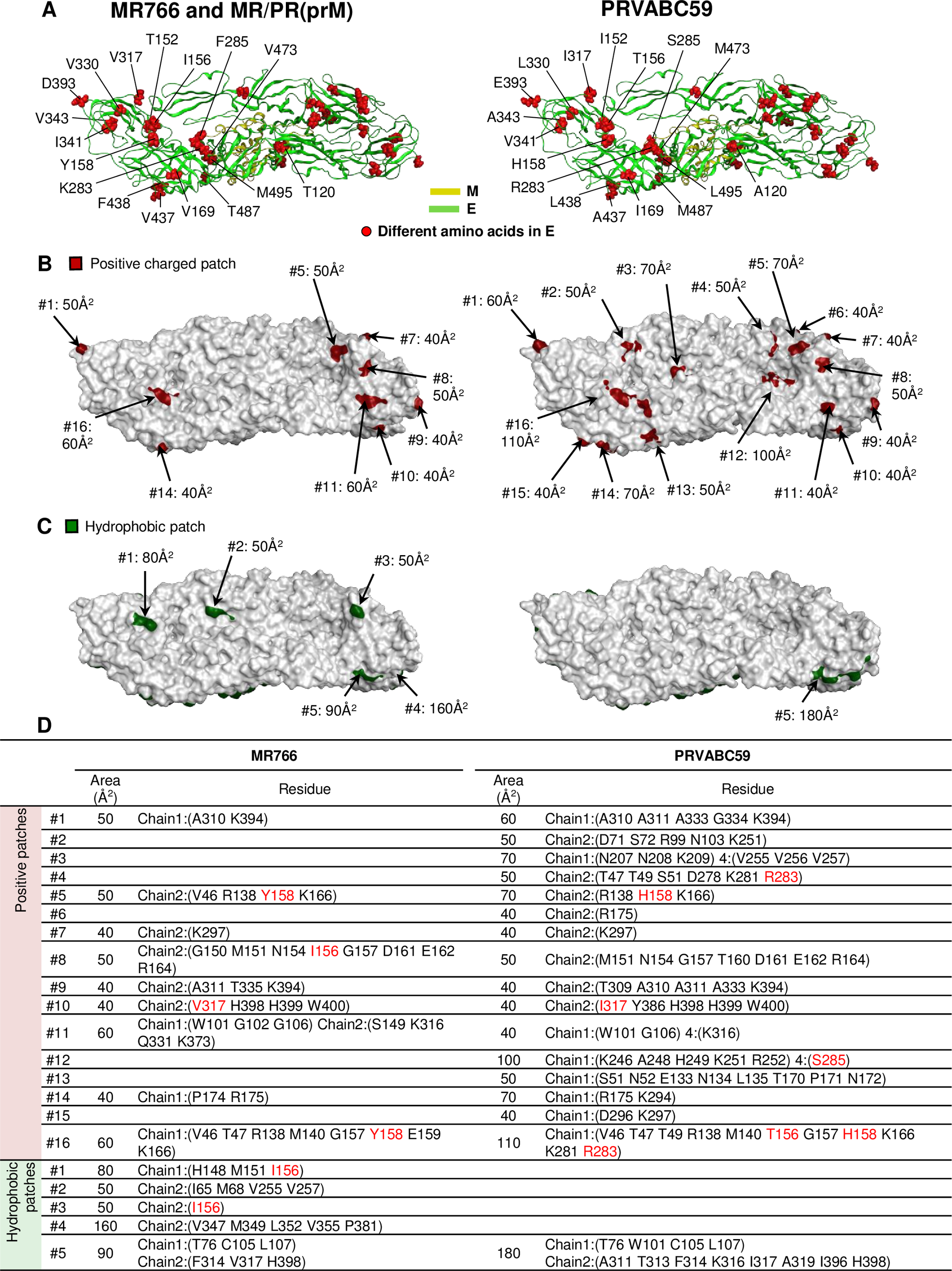

Supplement: S9 Fig — (A) The structure of dimeric E-M complex in the mature virus. The homology models based on 5IZ7 were generated by MOE homology modeler. The M and E proteins are shown in yellow and green, respectively. The 17 different amino acids in the E protein between MR766 and PRVABC59 are shown as red spheres. The positions of each different residue are indicated on one of the E proteins in the dimer. (B) The positively charged patch analysis shows the E protein of PRVABC59 has additional positively charged patches and an increase in the areas of other positively charged patches. These are due both to amino acid substitutions and slightly different folding and side chain exposures; see D below. The top view of the E dimers relative to the viral surface is shown. The threshold for a patch area was 50 Å2. The default MOE settings were used for protein surface patch analyses. (C) The hydrophobic patch analysis shows the E protein of PRVABC59 has fewer exposed hydrophobic patches. The top view of the E dimers relative to the viral surface is shown. The threshold and MOE settings as for B. (D) The amino acids contributing to each patch; substitutions highlighted in red. (TIF) [file ppat.1009788.s009.tif]

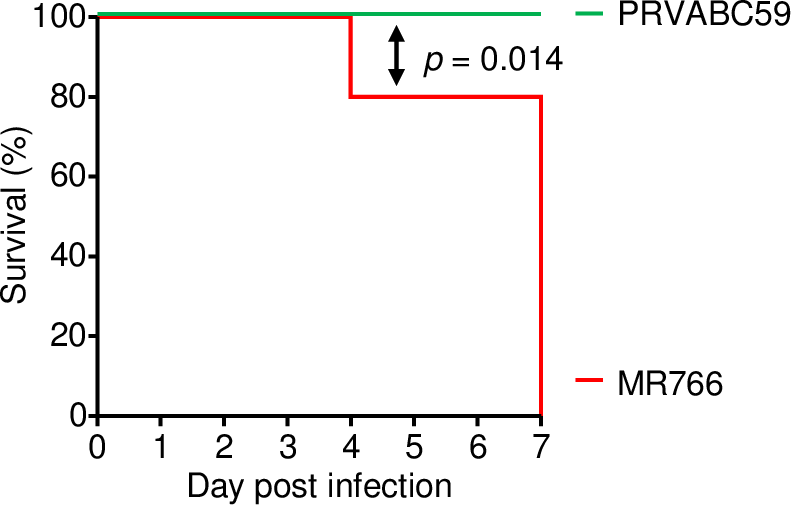

Supplement: S10 Fig — Neonatal C57BL/6J mice were infected i.c. with 1 × 10^4^ PFU of MR766 (n = 5) or PRVABC59 (n = 3) and monitored for 7 dpi. Comparison of Kaplan-Meier survival curves between groups was performed by log-rank analysis. (TIF) [file ppat.1009788.s010.tif]

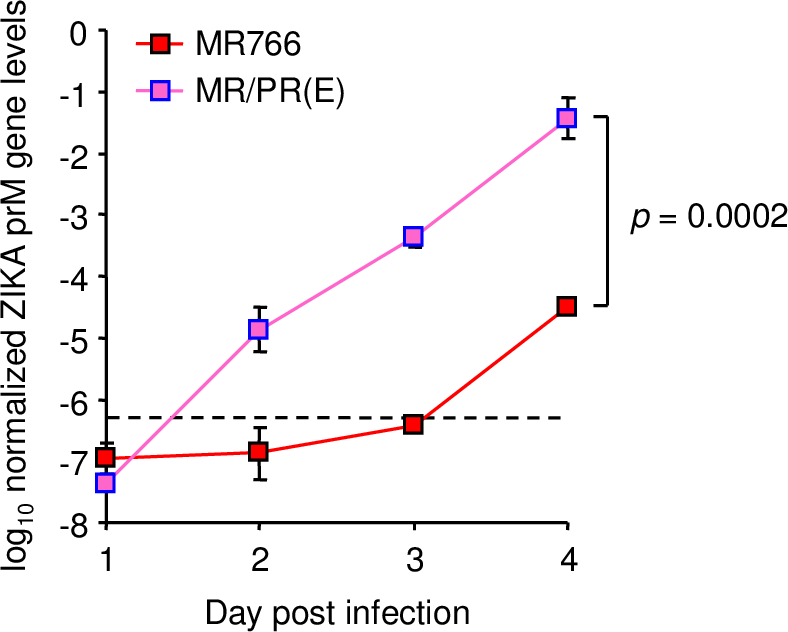

Supplement: S11 Fig — Ten to twelve-week-old IFNAR-/- mice were s.c. infected with 1 × 10^4^ PFU of MR766 or MR/PR(E) (n = 4–5 mice per group). At indicated time points, mice were sacrificed and perfused with 50 ml of cold PBS through the left ventricle of the heart to flush out intravascular viruses and the brains were collected. ZIKV RNA levels in brains were determined by qRT-PCR with the prM gene-specific primers and normalized to RPL13 mRNA levels. Three uninfected mouse brains were used to determine the limit of detection (10−6.29, indicated by the horizontal dashed line). Statistical analyses were performed using repeated-measures ANOVAs. (TIF) [file ppat.1009788.s011.tif]

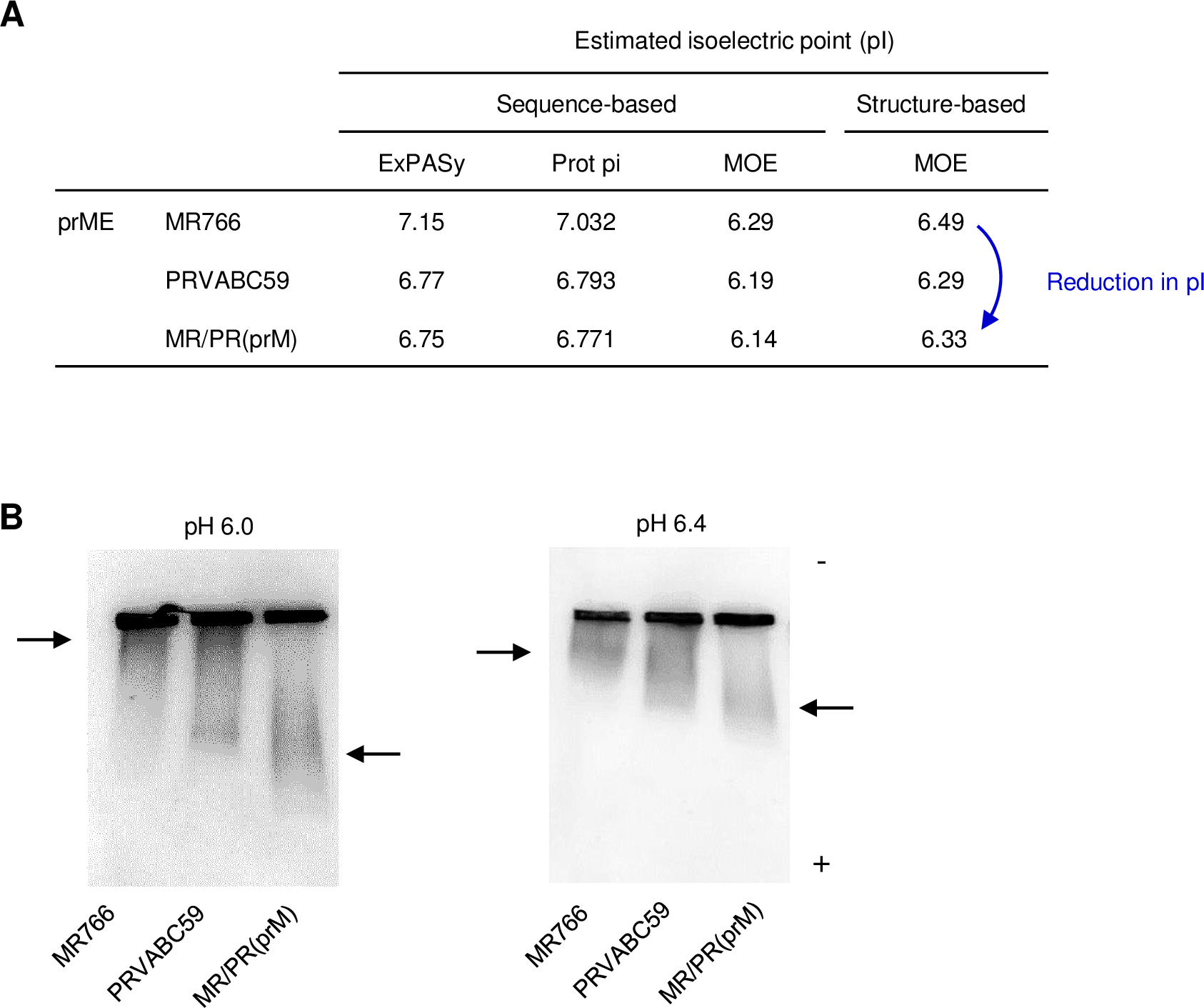

Supplement: S12 Fig — (A) The calculated pI value. The pI based on the sequences or protein structures was calculated using ExPASy ProtParam tool (http://www.expasy.ch/tools/protparam.html), Prot pi Protein Tool (https://www.protpi.ch/Calculator/ProteinTool) or MOE (Ver. 2019.01). In MOE, the sequence-based pI was calculated from the protein sequence according to [149] (see also http://isoelectric.org/www_old/files/practise-isoelectric-point.html) and structure-based pI was calculated using a modification to the algorithm of [149] in that individual amino acid group pKa’s estimated from 3D coordinates and local hydrogen bond networks are used. The pKa values are calculated according to the PROPKA algorithm [150]. The default MOE settings were used for the pI calculation. All three tools suggested that pI of the prME of MR766 was higher than the prME of MR/PR(prM). (B) Gel electrophoresis of purified MR766, PRVABC59 and MR/PR(prM) viruses in pH 6.0 or pH 6.4 buffers. The electrophoresis was performed as previously described [112], with modifications. Briefly, the surface of the 8 × 4 cm GelBond File (Lonza, Tokyo, Japan) was coated with 7–8 ml of 0.8% agarose (Takara Bio Inc. Shiga, Japan). The gels were electrophoresed in citric acid phosphate buffer (pH 6.0 or pH 6.4) [54] at 90 volts (ATTA model AE-8750) for 60 min at 4°C. After the electrophoresis, proteins were stained with Coomassie brilliant blue. Arrows indicate the position of MR766 or MR/PR(prM). (TIF) [file ppat.1009788.s012.tif]

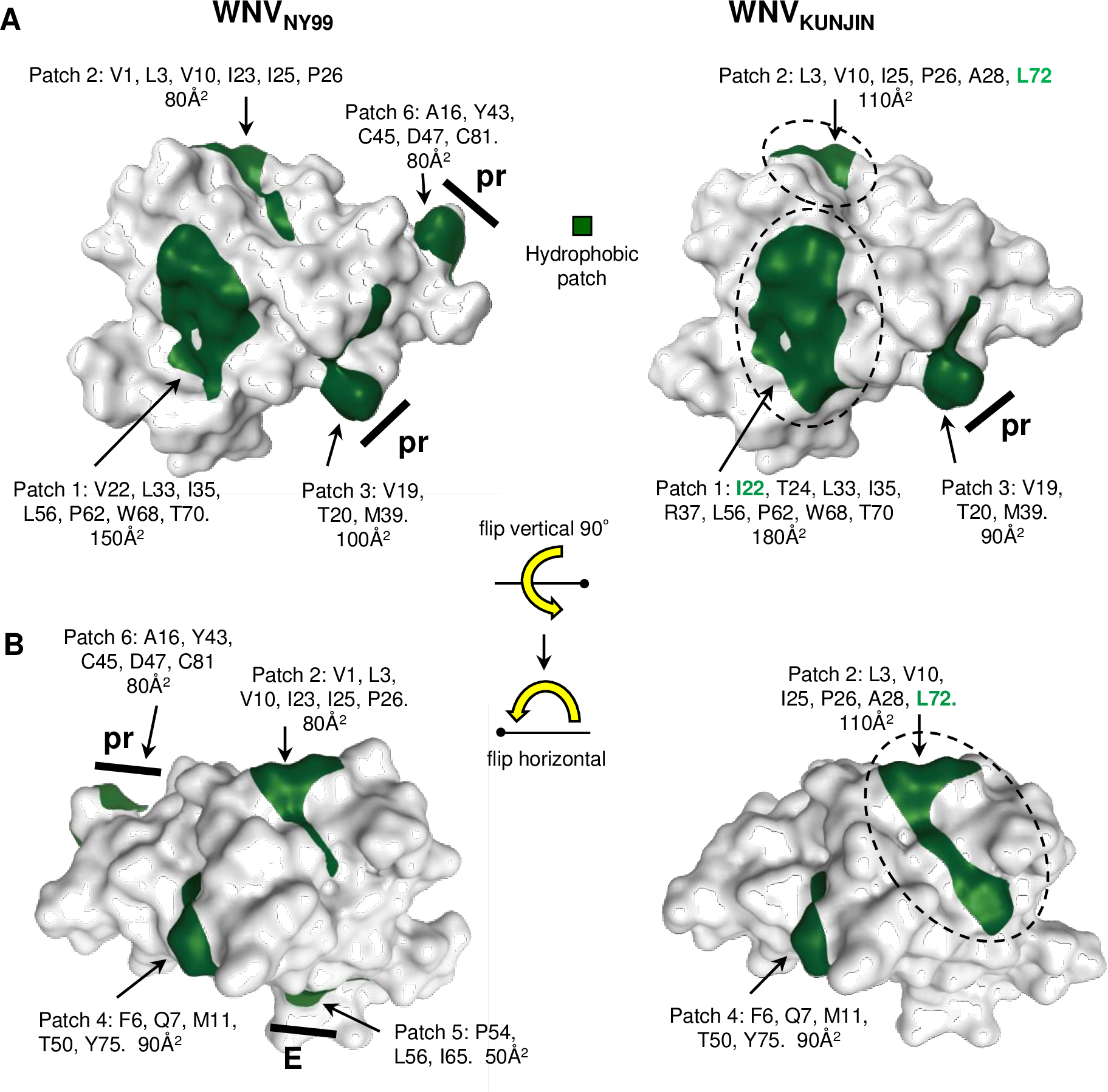

Supplement: S13 Fig — The homology model of WNVNY99 and WNVKUNJIN pr proteins based on the structure (PDB accession code: 3C6E) was generated with MOE homology modeler. Protein surface patch analysis shows that the pr protein of the WNVKUNJIN has larger hydrophobic patches (Patch 1 and Patch 2) (dashed ovals) exposed on the top of the trimeric spike. The increase in hydrophobic patch area was 60Å2 or 26% (80 + 150 versus 110 + 180). Patches 3 and 6 face other pr proteins, and Patch 5 faces the E protein in the trimeric spike. The 2 different amino acid residues (positions 22 and 72) in the pr protein between WNVNY99 and WNVKUNJIN are colored in green. The threshold of the patch area was 50Å2. The default MOE settings were used for protein surface patch analysis. (A) Top view or (B) side view of pr protein relative to the virion surface. (TIF) [file ppat.1009788.s013.tif]

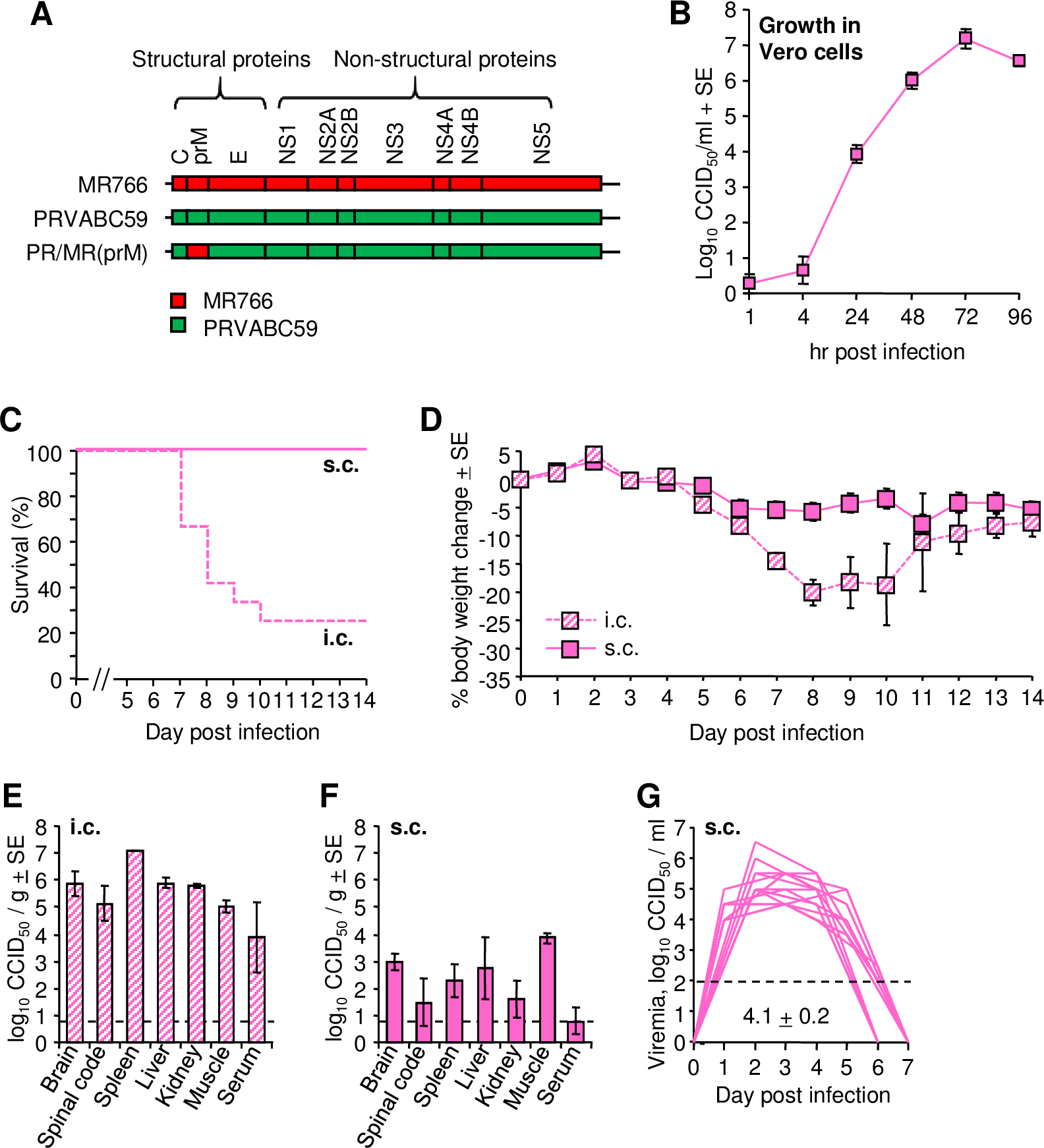

Supplement: S14 Fig — (A) Schematic representation illustrating prM gene swapping to generate PR/MR(prM). Recombinant molecular clones of PR/MR(prM) were constructed using the modified infectious-subgenomic-amplicons method and primers (see S1 Table) as previously described [151]. (B) Growth kinetics of PR/MR(prM) in Vero cells. Vero cells were infected at a MOI of 0.01. The viral titers were determined by CCID50 assays on Vero cells. Each data point represents the mean titer of 4 wells. (C) Survival of adult IFNAR-/- mice infected with 1 × 10^4^ PFU of PR/MR(prM) by i.c. (n = 11) or s.c. (n = 11) infection. The i.c. or s.c. infection with 1 × 10^4^ PFU of PR/MR(prM) resulted in 27.3% or 100% survival in IFNAR-/- mice, respectively. These survival data were not significantly different from PRVABC59 (data from Figs 2C and 4D): 41.7% (p = 0.62) or 100% (p = 1.00) survival after i.c. or s.c. infection, respectively. However, these survival data were significantly different from MR766 (data from Figs 2C and 4D): 0% (p<0.0001) for both i.c. and s.c. infections (by log-rank analysis). (D) Mean percent weight change relative to day 0 after infection with 1 × 10^4^ PFU of PR/MR(prM) by i.c. (n = 11) or s.c. (n = 11). (E) Tissue titers after i.c. infection with 1 × 10^4^ PFU of PR/MR(prM) (n = 4). Organs were harvested at 4 dpi, and viral titers were determined by CCID50 assays. The brain viral titer for PR/MR(prM)-infected mice was 2.4 logs lower than that for MR766 (p = 0.0004 by t-test, Fig 4C). Limit of detection was 0.83 log10CCID50/g indicated by the horizontal dashed line. (F) As in E after s.c. infection with 1 × 10^4^ PFU of PR/MR(prM) (n = 5). Organs were harvested at 6 dpi. (G) Viremia of individual IFNAR-/- mice infected s.c. with 1 × 10^4^ PFU of PR/MR(prM) (n = 11). These viremias levels for 1–7 dpi were not significantly different from those obtained after s.c. infection with 1 × 10^4^ PFU of MR766 or PRVABC59 (data from S2B Fig) (p = 0.69 or p = 0.34, respectively; repeated-measures ANOVA). Lim [file ppat.1009788.s014.tif]

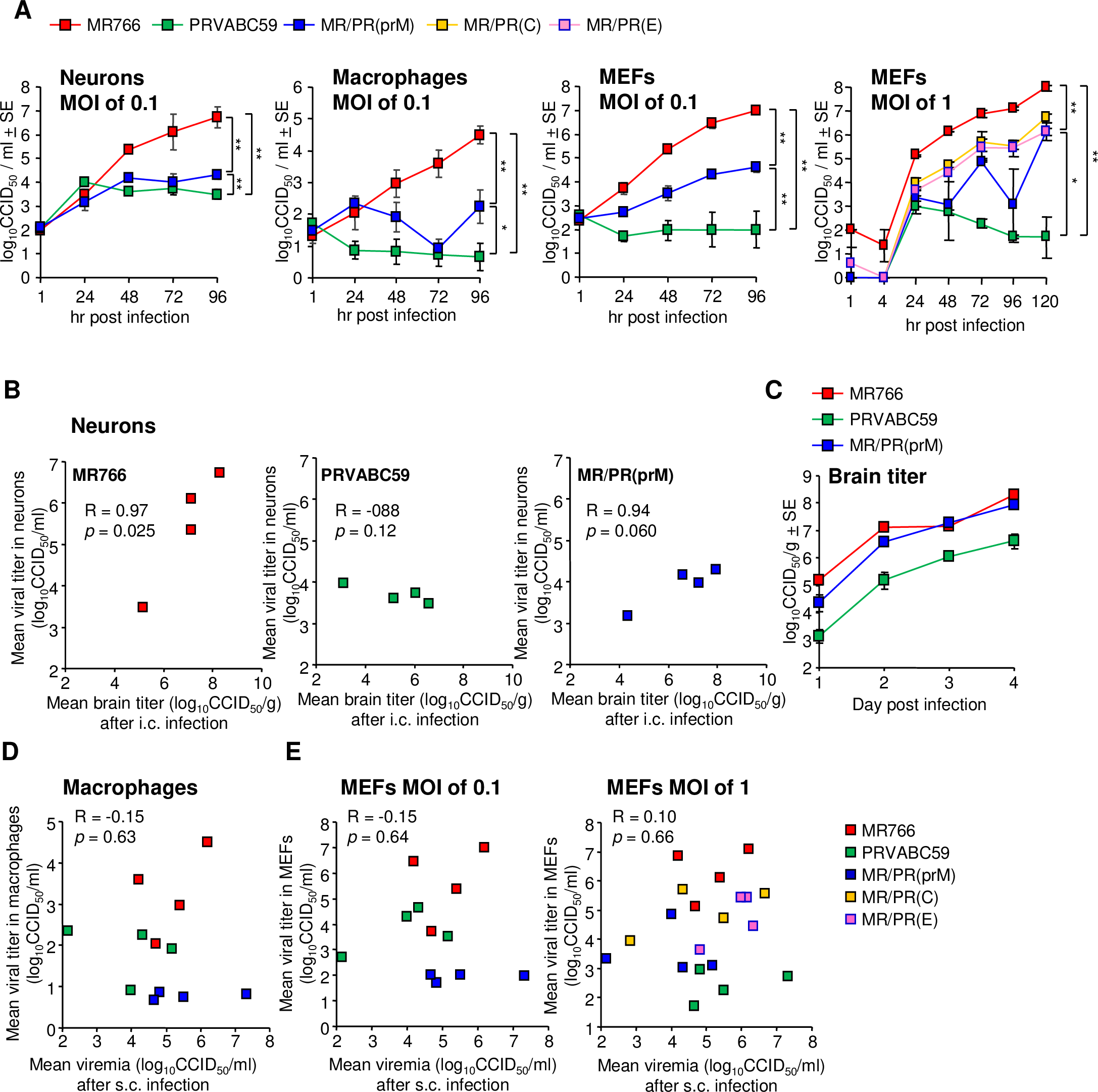

Supplement: S15 Fig — (A) Primary neurons, macrophages and MEFs were infected at a MOI of 0.1 or 1 and the supernatants were collected at the indicated times. The viral titers were determined by CCID50 assays on Vero cells. Each data point represents the mean titer of 3–7 wells. Statistical analyses were performed using repeated-measures ANOVAs: * p < 0.05; ** p < 0.01. (B) The correlation diagrams between mean viral titer in neurons and mean brain titer after i.c. infection. Ten to fourteen-week-old IFNAR-/- mice were i.c. infected with 1 × 10^4^ PFU of MR766, PRVABC59 or MR/PR(prM) and the brains were harvested at 1, 2 or 3 dpi (n = 4–5 mice per group). The viral titers were determined by CCID50 assays. The brain titers at 4 dpi are the same as those shown in Fig 4C. Significance was determined by Pearson’s correlation test. (C) Viral titers in brains after i.c. infection. (D) No significant correlation between mean viral titer in macrophages and mean viremia after s.c infection. Significance was determined by Pearson’s correlation test. (E) No significant correlation between mean viral titer in MEFs and mean viremia after s.c. infection. Significance was determined by Pearson’s correlation test. (TIF) [file ppat.1009788.s015.tif]

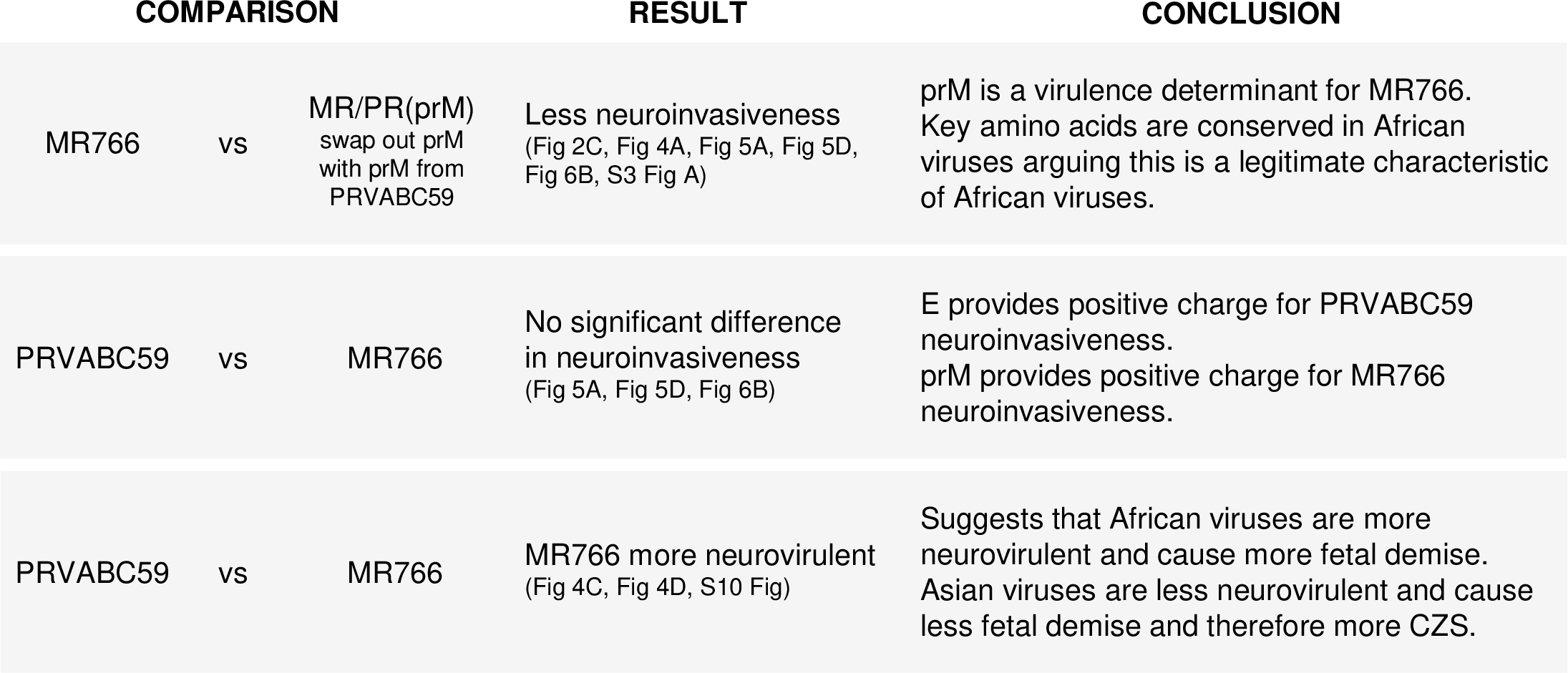

Supplement: S16 Fig — (TIF) [file ppat.1009788.s016.tif]
