# Supplementary material for: Neuroinvasiveness of the MR766 strain of Zika virus in IFNAR-/- mice maps to prM residues conserved amongst African genotype viruses
Source: PLoS Pathog. 2021 Jul 26;17(7):e1009788. doi: 10.1371/journal.ppat.1009788 (PMC8341709; doi:10.1371/journal.ppat.1009788)
Supplement: S1 Table — References: (1) Lanciotti RS, Kosoy OL, Laven JJ, Velez JO, Lambert AJ, Johnson AJ, et al. Genetic and serologic properties of Zika virus associated with an epidemic, Yap State, Micronesia, 2007. Emerg Infect Dis. 2008;14:1232–39; (2) Dang JW, Tiwari SK, Qin Y, Rana TM. Genome-wide Integrative Analysis of Zika-Virus-Infected Neuronal Stem Cells Reveals Roles for MicroRNAs in Cell Cycle and Stemness. Cell Rep. 2019;27(12):3618–28 e5; (3) Gardner J, Anraku I, Le TT, Larcher T, Major L, Roques P, et al. Chikungunya virus arthritis in adult wild-type mice. J Virol. 2010;84:8021–32; (4) Castro-Jorge LA, Pretto CD, Smith AB, Foreman O, Carnahan KE, Spindler KR. A Protective Role for Interleukin-1 Signaling during Mouse Adenovirus Type 1-Induced Encephalitis. J Virol. 2017;91:e02106-16; (5) Wang P, Dai J, Bai F, Kong KF, Wong SJ, Montgomery RR, et al. Matrix metalloproteinase 9 facilitates West Nile virus entry into the brain. J Virol. 2008;82:8978–85. (DOCX) [file ppat.1009788.s017.docx]

**S1 Table. Primers used in the study.**

**Primers for qPCR**

| Target | Forward primer (5′–3′) | Reverse primer (5′–3′) | Reference |
| --- | --- | --- | --- |
| ZIKV E | CCGCTGCCCAACACAAG | CCACTAACGTTCTTTTGCAGACAT | (1) |
| PRVABC59 prM | TTGGTCATGATACTGCTGATTGC | CCTTCCACAAAGTCCCTATTGC | (1) |
| MR766 prM | TTGGTCATGATACTGCTGATTGC | CCCTCCACGAAGTCTCTATTGC | (2) |
| Mouse RPL13A | GAGGTCGGGTGGAAGTACCA | TGCATCTTGGCCTTTTCCTT | (3) |
| Mouse TNF-α | CCACCACGCTCTTCTGTCTAC | AGGGTCTGGGCCATAGAACT | (4) |
| Mouse IL-1β | GCAACTGTTCCTGAACTCAACT | ATCTTTTGGGGTCCGTCAACT | (4) |
| Mouse IL-6 | CCAGAAACCGCTATGAAGTTCC | TCACCAGCATCAGTCCCAAG | (5) |

**Primers to generate chimeric ZIKVs**

| Chimera name | Primer name | Sequence (5′–3′) |
| --- | --- | --- |
| MR/PR(C) | MRPR120.r | TTTGGGTTTTTCATGACCAGAAAC |
|  | MRPR106.f | CATGAAAAACCCAAAAAAGAAATC |
|  | PR.445.r | TGCCATAGCTGTGGTCAGCAG |
|  | MP.C.Vec.f | ACCACAGCTATGGCAGCAGAGATCACTAGACGCGG |
| MR/PR(prM) | MP.prM.Vec.r | CTAGTGACCTCCGCTGCCATGGCTGTAGTCAGCAG |
|  | PR.445.f | AGCGGAGGTCACTAGACGTGGG |
|  | PR949.r | GCTGTATGCCGGGGCAATCAG |
|  | MPprM.Vec.f | GCCCCGGCATACAGCATCAGGTGCATTGGAGTCAG |
| MR/PR(E) | MP.E.Vec.r | TCCTATGCACCTGATACTGTATGCCGGGGC |
|  | PR.950.f | ATCAGGTGCATAGGAGTCAGC |
|  | PR.2461.r | AGCAGAGACGGCTGTGGATAAG |
|  | MP.E.Vec.f | ACAGCCGTCTCTGCTGACGTGGGGTGCTCAGTGGA |
| MR/PR(NS4B/NS5) | ZKV.7642(NS4B).r2 | TCTCGTCACTGTATAGATAAGG |
|  | PR.NS5.MR.IF.f2 | TATACAGTGACGAGAAACGCTGGCTTGGTCAAGAG |
|  | PR.NS5.MR.IF.r | TAAAATTGGTGCTTACAGCACTCCAGGTGTAGACC |
|  | ZKV.10376(3NCR).f | TAAGCACCAATTTTAGTGTTG |
| MR/PR(NS5) | ZKV.7666(NS4B).r | ACGTCTCTTAACCAGGCCAGC |
|  | PR.NS5.MR.IF.f | CTGGTTAAGAGACGTGGGGGTGGAACAGGAGAGAC |
|  | PR.NS5.MR.IF.r | TAAAATTGGTGCTTACAGCACTCCAGGTGTAGACC |
|  | ZKV.10376(3NCR).f | TAAGCACCAATTTTAGTGTTG |
| MR766-NIID-NDT | m231.f | AATGATACAGGATATGAAACTGACGAAAATAG |
|  | m231.r | ATATCCTGTATCATTGACAGTCATCCCGCTATG |
| MR766-NIID-Δ153-156 | ZIKA.1442.f | ATAGGATATGAAACTGACGAAAA |
|  | ZIKA.1429.r | CATCCCGCTATGCTGGGAGC |
| MR766-NIID-Δ156-161 | ZKV.Fdel.IF.f | CAATGATGAAAATAGAGCGAAAGTCGAG |
|  | ZKV.Fdel.IF.R | CTATTTTCATCATTGACAGTCATCCCGCTATG |
| PR/MR(prM) | MR/pr_1F | AGCAGAGATCACTAGACGCGGGAG |
|  | MR/M_225R | ACTGTATGCCGGGGCAATCAG |
|  | PR/C_MR/pr_R | CTAGTGATCTCTGCTGCCATAGCTGTGGTCAGCAG |
|  | MR/M_PR/E_F | GCCCCGGCATACAGTATCAGGTGCATAGGAGTCAG |
| MR/PR(pr) | MR/C_366R | TGCCATGGCTGTAGTCAGCAGGAGG |
|  | MR/M_1F | GCCGTGACGCTCCCTTCTCACTCTA |
|  | MR/C_PR/pr_F | ACTACAGCCATGGCAGCGGAGGTCACTAGACGTGG |
|  | PR/pr_MR/M_R | AGGGAGCGTCACGGCTCTTCTAGATCTCCGTGCTT |
| MR/PR(M) | MR/pr_279R | TCTTCTAGATCGCCGTGCCTCACC |
|  | MR/E_1F | ATCAGGTGCATTGGAGTCAGCAATA |
|  | MR/pr_PR/M_F | CGGCGATCTAGAAGAGCTGTGACGCTCCCCTCCCA |
|  | PR/M_MR/E_R | TCCAATGCACCTGATGCTGTATGCCGGGGCAATCA |
