# Supplementary material for: Neuroinvasiveness of the MR766 strain of Zika virus in IFNAR-/- mice maps to prM residues conserved amongst African genotype viruses
Source: PLoS Pathog. 2021 Jul 26;17(7):e1009788. doi: 10.1371/journal.ppat.1009788 (PMC8341709; doi:10.1371/journal.ppat.1009788)
Supplement: S2 Table — The ASA(S) of each amino acid was calculated with the ASA Calculator program (MOLSIS Inc., Tokyo, Japan) operated by MOE, using the homology model of the trimer structure of prME (PDB accession code: 5U4W). Solvent accessibility classes were defined relative to values calculated for all residue types in a Gly-X-Gly tripeptide (both backbone and side-chain conformations of X being fully extended). Three solvent accessibility classes are defined: buried (< 9%), partially exposed (< 36%) and exposed (> 36%) [93]. Color coding as in Fig 7 (red–positively charged, green–hydrophobic patch). (DOCX) [file ppat.1009788.s018.docx]

**S2 Table.** **The solvent-accessible surface area of amino acid side-chains (ASA(S)) of the seven amino acids that differ between the pr proteins of MR766 and MR/PR(prM).**

| Chain | Position | MR766 | | | MR/PR(prM) | | |
| --- | --- | --- | --- | --- | --- | --- | --- |
|  |  | **Residue** | **ASA(S)**  **(Å^2^)** | **Ratio**  **(%)** | **Residue** | **ASA(S)**  **(Å^2^)** | **Ratio**  **(%)** |
| pr1 | 3 | I | 32.09 | 25 | V | 30.71 | 30 |
|  | 17 | S | 84.64 | 100 | N | 91.53 | 77 |
|  | 21 | K | 76.66 | 47 | E | 103.11 | 75 |
|  | 26 | A | 56.53 | 100 | P | 89.47 | 100 |
|  | 31 | V | 75.95 | 74 | M | 66.95 | 47 |
|  | 35 | H | 71.39 | 50 | Y | 68.44 | 36 |
|  | 36 | V | 0 | 0 | I | 0 | 0 |
| pr2 | 3 | I | 35.58 | 27 | V | 31.11 | 30 |
|  | 17 | S | 71.59 | 97 | N | 83.68 | 71 |
|  | 21 | K | 63.53 | 39 | E | 87.54 | 63 |
|  | 26 | A | 59.64 | 100 | P | 84.90 | 98 |
|  | 31 | V | 74.26 | 73 | M | 64.09 | 45 |
|  | 35 | H | 63.13 | 44 | Y | 71.40 | 38 |
|  | 36 | V | 0 | 0 | I | 0 | 0 |
| pr3 | 3 | I | 35.78 | 28 | V | 30.45 | 30 |
|  | 17 | S | 82.61 | 100 | N | 98.08 | 0.83 |
|  | 21 | K | 75.45 | 46 | E | 117.22 | 85 |
|  | 26 | A | 59.64 | 100 | P | 83.88 | 97 |
|  | 31 | V | 77.53 | 76 | M | 68.36 | 48 |
|  | 35 | H | 62.34 | 44 | Y | 66.15 | 35 |
|  | 36 | V | 0 | 0 | I | 0 | 0 |
